# HPV E6 inhibits E6AP to regulate epithelial homeostasis by modulating keratinocyte differentiation commitment and YAP1 activation

**DOI:** 10.1371/journal.ppat.1011464

**Published:** 2023-06-28

**Authors:** Wen Yin, Nagayasu Egawa, Ke Zheng, Heather Griffin, Pu Tian, Ademola Aiyenuro, Jacob Bornstein, John Doorbar

**Affiliations:** 1 Department of Pathology, University of Cambridge, Cambridge, United Kingdom; 2 Gynecologist & Obstetrician, Colposcopy, Azrieli Faculty of Medicine of Bar-Ilan University, and Galilee Medical Center–Nahariya; University of Wisconsin Madison School of Medicine and Public Health, UNITED STATES

## Abstract

Human papillomaviruses (HPV) cause persistent infections by modulating epithelial homeostasis in cells of the infected basal layer. Using FUCCI and cell-cell competition assays, we have identifed regulatory roles for E6AP and NHERF1, which are the primary HPV11 E6 cellular targets, as well as being targets of the high-risk E6 proteins, in processes governing epithelial homeostasis (i.e. cell density, cell cycle entry, commitment to differentiation and basal layer delamination). Depletion of E6AP, or expression of HPV11 or 16E6 increased keratinocyte cell density and cell cycle activity, and delayed the onset of differentiation; phenotypes which were conspicuously present in HPV11 and 16 infected patient tissue. In line with proposed E6 functions, in HPV11 condyloma tissue, E6AP and NHERF1 were significantly reduced when compared to uninfected epithelium. In experimental systems, loss of HPV11 E6/E6AP binding abolished 11E6’s homeostasis regulatory functions, while loss of E6/NHERF1 binding reduced the cell density threshold at which differentiation was triggered. By contrast, a NHERF1-binding mutant of 16E6 was not compromised in its homeostasis functions, while E6AP appeared essential. RNA sequencing revealed similar transcriptional profiles in both 11 and 16E6-expressing cells and E6AP^-/-^ cells, with YAP target genes induced, and keratinocyte differentiation genes being downregulated. HPV11 E6-mediated Yap activation was observed in 2D and 3D (organotypic raft) cell culture systems and HPV-infected lesions, with both NHERF1, which is a regulator of the Hippo and Wnt pathways, and E6AP, playing an important role. As the conserved binding partner of Alpha group HPV E6 proteins, the precise role of E6AP in modulating keratinocyte phenotype and associated signalling pathways has not previously been defined. Our study suggests a model in which the preserved functions of the low and high-risk Alpha E6 proteins modulate epithelial homeostasis via E6AP activity, and lead to alteration of multiple downstream pathways, including those involving NHERF1 and YAP.

## Author summary

Human papillomaviruses are classified as ‘high-risk’ and ‘low-risk’ types. Both groups of viruses establish a reservoir of infection in the epithelial basal layer. To maintain persistence and support the productive lifecycle, early viral proteins such as E6 interact with cellular targets to modulate the epithelial homeostasis pathways. Here we have used a cell cycle indicator system, together with cell-cell competition assays to assess the functions of HPV E6 protein in keratinocytes. We found that both ‘low-risk’ and ‘high risk’ E6 proteins drive cell proliferation, and delay cell contact inhibition, delamination and differentiation in our model system, which are the four necessary steps to maintain the balance of basal cell expansion and loss. We identified that cellular proteins E6AP and NHERF1 both contribute to E6 functions, and discovered that E6AP itself also regulates keratinocyte differentiation. The key downstream pathways regulated by E6/E6AP include the Hippo and Wnt pathways. Its activation is known to promote cell proliferation and inhibit cell differentiation. Therefore, we propose a model in which E6 activates YAP activity by inhibiting E6AP and NHERF1 in the basal layer to support homeostasis. These findings will enhance our understanding of the nature of chronic lesions and self-limiting HPV-associated diseases.

## Introduction

Human papillomaviruses (HPV) are non-enveloped double-stranded DNA viruses that infect multiple epithelial sites[1,2]. So far, more than 200 HPV types based on L1 viral gene sequence identity have been discovered, which are classified into five genera: Alpha-, Beta-, Gamma, Mu and Nu-papillomaviruses [3]. The Alpha genus is comprised of viruses that can either infect cutaneous or mucosal epithelial sites, those mucosal HPVs can be further classified into high-risk and low-risk HPVs based on the cancer risk associated with their infections[2,4]. HPV16 and HPV11 are representatives of the Alpha high-risk group and the low-risk group. HPVs generally cause self-limiting epithelial lesions that are usually resolved by the host immune system over time. However, the high-risk mucosal HPV infections can sometimes persist and lead to carcinomas [5]. Although low-risk HPVs rarely cause malignancies, the size and location of the benign papillomas can render these lesions medically serious [6]. For example, recurrent respiratory papillomatosis (RRP) caused by HPV11 in children has no effective treatment and can only be controlled by repeat surgery. Condyloma acuminatum caused by HPV6 and HPV11 is one of the most widespread sexually transmitted diseases [7,8].

The epidermis is a stratified epithelium composed of morphologically distinct cellular layers that reflect the terminal differentiation process of keratinocytes [9]. Basal keratinocytes proliferate and expand to reach a specific density which triggers contact inhibition signals. This causes the cells to exit the cell cycle and to commit to differentiation. Subsequently, keratinocytes leave the basal layer (delamination) and enter the more superficial spinous, granular, and cornified layers [10]. Epithelial homeostasis is maintained by the careful regulation of cell cycle entry, basal cell density, delamination and differentiation. HPV viral proteins impart advantages to infected basal cells by modulating these key processes, leading to lesion expansion and subsequent lesion maintenance [11]. Previous literature has identified E6 as a homeostasis regulator during productive infection, and in both the high and low-risk Alpha types, can regulate p53 and thus indirectly Notch-mediated epithelial differentiation[12–15]. In fact, accumulating evidence suggests that E6 modulates epithelial homeostasis through interaction with key effector proteins involved in the Wnt, Notch and Hippo signalling pathways [16–19]. The Hippo pathway effector yes-associated protein (YAP) plays a pivotal role in controlling epidermal homeostasis, with the high-risk E6 protein shown to control the YAP cytoplasm-nucleus shift in order to activate YAP transcriptional activity [20,21]. The Hippo pathway senses mechanical cues such as cell density and contact signals from the basement membrane. Once activated, YAP is phosphorylated by LATS1/2 kinases and sequestered in the cytoplasm. When the Hippo kinases are inactive, YAP is translocated into the nucleus and activates the downstream genes to drive keratinocyte proliferation[21,22]. It has also been shown that the inhibition of YAP activity triggers keratinocyte differentiation[23,24].

It has been reported previously that the Alpha genus E6 proteins are the only group that preferentially interacts with E6AP rather than MAML1, with some E6 proteins further acquiring the ability to induce E6AP degradation[25]. E6 is typically associated with E6AP to form a complex that recruits secondary substrates such as p53 or NHERF1 for proteasomal degradation[25–27]. Indeed, we believe that these cellular targets of E6 may be important regulatory factors involved in epithelial homeostasis control. As the conserved binding partner of the Alpha genus E6 proteins, E6AP plays a central role, with the consequences of E6AP association varying among E6 proteins. The association of E6 with E6AP for instance, does not necessarily trigger its ubiquitination activity [25]. Similarly, the downstream consequences and significance of E6AP degradation by E6 are poorly understood. E6AP has been reported to regulate Wnt signalling, with this function being intensified by E6 in primary keratinocytes [19]. Also, E6AP was found to promote cell cycle entry in multiple cell types [28–30], indicating a potential role for E6AP in modulating homeostasis functions in its own right. NHERF1 was one of the first cellular targets discovered to be degraded by both high- and low-risk E6-E6AP complex, and has been shown to interact with β-catenin and YAP directly[31,32]. Also, it was demonstrated that NHERF1 degradation by 16E6 leads to the activation of the Wnt pathway[25]. Thus, E6-directed NHERF1 degradation may also contribute to homeostasis regulation and lesion persistence in the epithelium.

Low-risk HPVs successfully survive and cause chronic lesions in the epithelium [33]. Thus, low-risk HPVs must possess the basic set of homeostatic functions to support their persistence in the basal epithelium [11]. It is anticipated that these functions are conserved across the Alpha papillomavirus genus and are important for maintaining the virus lifecycle. In this study, we have dissected the role of E6 in regulating aspects of epithelial homeostasis using normal spontaneously immortalised keratinocytes (NIKS) [34], primary keratinocytes and HPV-infected clinical biopsies. Our studies suggest that E6AP and NHERF1, which are common cellular targets of the E6 proteins of Alpha HPV types, are regulated by the virus in the epithelial basal layer during the HPV lifecycle. Using E6AP^-/-^ keratinocyte cell lines, our results further suggest that E6AP may function as a homeostasis modulator even in the absence of E6, with transcriptome analysis indicating a decline in differentiation-related gene expression in keratinocytes. Curiously, E6 expression or E6AP depletion activated a similar subset of YAP target genes involved in cell proliferation. Our results suggest that E6 regulates key homeostatic processes in the epithelial basal layer by inhibiting E6AP function, and that this is complemented in 11E6 expressing cells by the downregulation of NHERF1. We suspect that disruption of the homeostatic pathway may have detrimental effects on viral persistence and offers attractive targets for therapeutic approaches.

## Results

### 11E6 and 16E6 proteins modulate the balance between cell proliferation and differentiation

**Fig 1:**
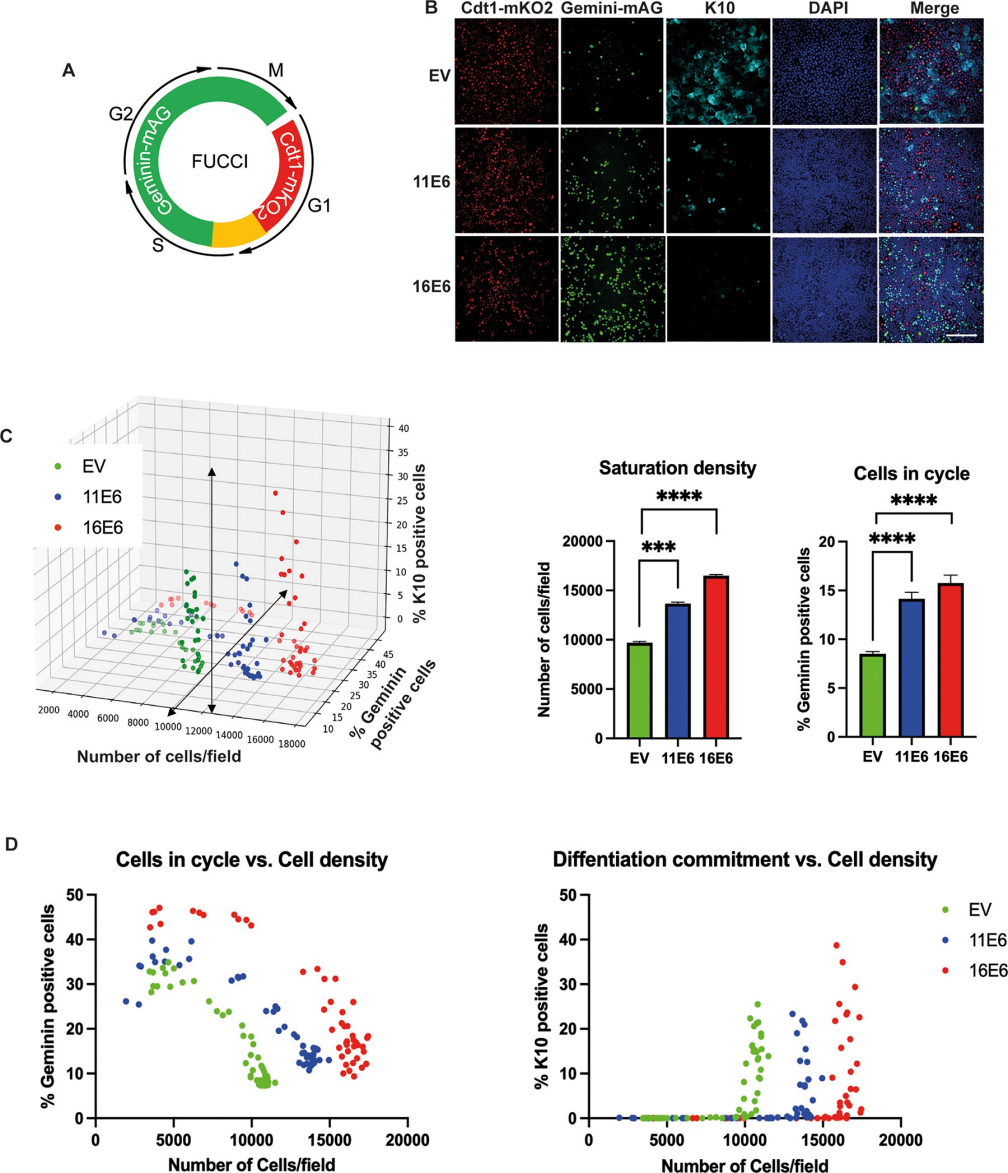
11E6 and 16E6 proteins regulate cell cycle progression, saturation density and differentiation of human keratinocytes. (A) Schematic diagram of the FUCCI system. (B) Typical field of view showing cells transduced with empty vectors (EV) or plasmids stably expressing either 11E6 or 16E6. At similar densities (10,000 cells/field), the expression of 11E6 or 16E6 increased geminin positive cells and decreased K10 expression. Red: Cdt1-mKO2 (G1 phase); Green: Geminin-mAG (S/G2/M phase); Blue: DAPI; Cyan: K10. (Original magnification, x20. Scale bar = 200 μm) (C) Three-dimensional graph displaying % geminin-positive cells, % K10 positive cells against number of cells per field in EV-transduced, 11E6-expressing or 16E6-expressing NIKS cell populations. Each data point is a 3.78 mm^2^ field of view. Significant differences of the cell saturation density and % cells in cycle at saturation across three cell populations were presented by the graphs on the right. Kruskal-Wallis tests were performed with Dunn’s correction. ***, *P* ≤0.001; ****, *P* ≤0.0001. (D) 2D images showing the effect of E6 expression on cells in cycle vs. cell density and differentiation commitment vs. cell density.

Recent work has demonstrated the role of E6 proteins in regulating several aspects of homeostasis in keratinocytes. This includes E6-enhanced keratinocyte proliferation, E6-mediated inhibition of keratinocyte differentiation, as well as the effect on cell-cell contact inhibition [13,14,35–37]. To maintain basal layer homeostasis, four processes need to be precisely controlled: cell cycle entry and proliferative potential, cell density, and the timing of delamination and differentiation [38]. To monitor the impact of E6 on cell cycle status, the fluorescent ubiquitination-based cell cycle indicator (FUCCI) system was used [39,40]. FUCCI relies on the phase-dependent proteolysis of the oscillators Cdt1 and geminin, and it is a powerful tool for visualizing cell cycle progression (Fig 1A). By combining this system with use of the differentiation marker K10 and staining with DAPI, it is possible to examine three of the four homeostasis-related phenotypes; i.e. cell cycle activity, differentiation commitment and saturation density. Preliminary results using this system indicated that when FUCCI-expressing NIKS cells transduced with an empty vector (NIKS-EV) reached post-confluence, there were more Cdt1-mKO2 positive cells (red) and fewer geminin-mAG positive cells (green) than in E6-expressing NIKS cells at the same cell density (Fig 1B), which fits with our understanding of E6-mediated cell proliferation upon contact inhibition. Importantly, NIKS-EV displayed higher K10 expression than NIKS expressing either 11E6 or 16E6, suggesting that in the absence of E6, keratinocytes become committed to differentiation at a lower cell density threshold than when E6 is present. To examine this further, NIKS keratinocytes were plated over a range of cell densities, from pre- to post-confluence. Cells were left to adhere, form contacts and to grow for 72 hours after plating, and then fixed and scanned by Confocal microscopy. High content imaging allowed quantification of the number of cells (saturation density), % K10-positive cells (differentiation commitment), and % geminin-mAG-positive cells (cells in cycle) for each field within the 96-well plates (Fig 1C). These three inter-linked phenotypes were plotted on a single graph, to reveal the differences among cell populations that are due to E6 expression. In ‘normal’ keratinocytes (i.e. not expressing E6), contact inhibition is triggered as cell density increases, with cells then enter G1 or G0 phase prior to differentiation [10,41]. Thus, the percentage of geminin-positive cells decreased as cell density increased. As shown in Fig 1D, the number of geminin-positive NIKS-EV cells declined from 32.6% to 7% and reached the steady-state levels where cell density in the dish remained constant. The expression of K10 occurred once saturation density was reached at approximately 10,000 cells per field (Fig 1D). In comparison to NIKS-EV cells, the percentage of geminin-positive cells dropped from 37.7% at pre-confluence to 12% at post-confluence when 11E6 was expressed. Geminin-positive cells decreased from 46.4% to 14% following 16E6 expression. Thus, an overall increase in cell cycle was observed both pre- and post-confluence in NIKS expressing either 11E6 or 16E6. Additionally, the expression of 11E6 or 16E6 increased the cell saturation density of the monolayer to approximately 14,000 cells or 17,000 cells/field respectively, and delayed the density threshold at which keratinocyte differentiation is triggered. A similar trend, albeit with a lower magnitude change, was also observed in normal human epidermal keratinocytes (NHEK) stably expressing either 11E6 or 16E6 (S1 Fig). Although we were unable to stably propagate HPV11 genomes in keratinocytes grown *in vitro*, this is possible for HPV16. Organotypic rafts expressing 16E6 and HPV16 whole genome (S2 Fig) showed an increase in ‘basal’ cell density, a small but measurable delay in the timing of K10 expression (differentiation committment), and a prominent increase in % of MCM-positive cells (cell in cycle) in the basal layer. These studies support the idea that E6 plays a role in regulating homeostasis when expressed alone and in the context of whole genome, but points to a role for additional viral proteins in regulating the onset of differentiation. Overall, our results suggest a role for E6 in modulating the cell density at which the homeostasis steady-state is achieved, and the time at which the infected cell transitions from proliferation to differentiation.

### E6AP and NHERF1 are crucial for E6 functions in cell proliferation and differentiation

**Fig 2:**
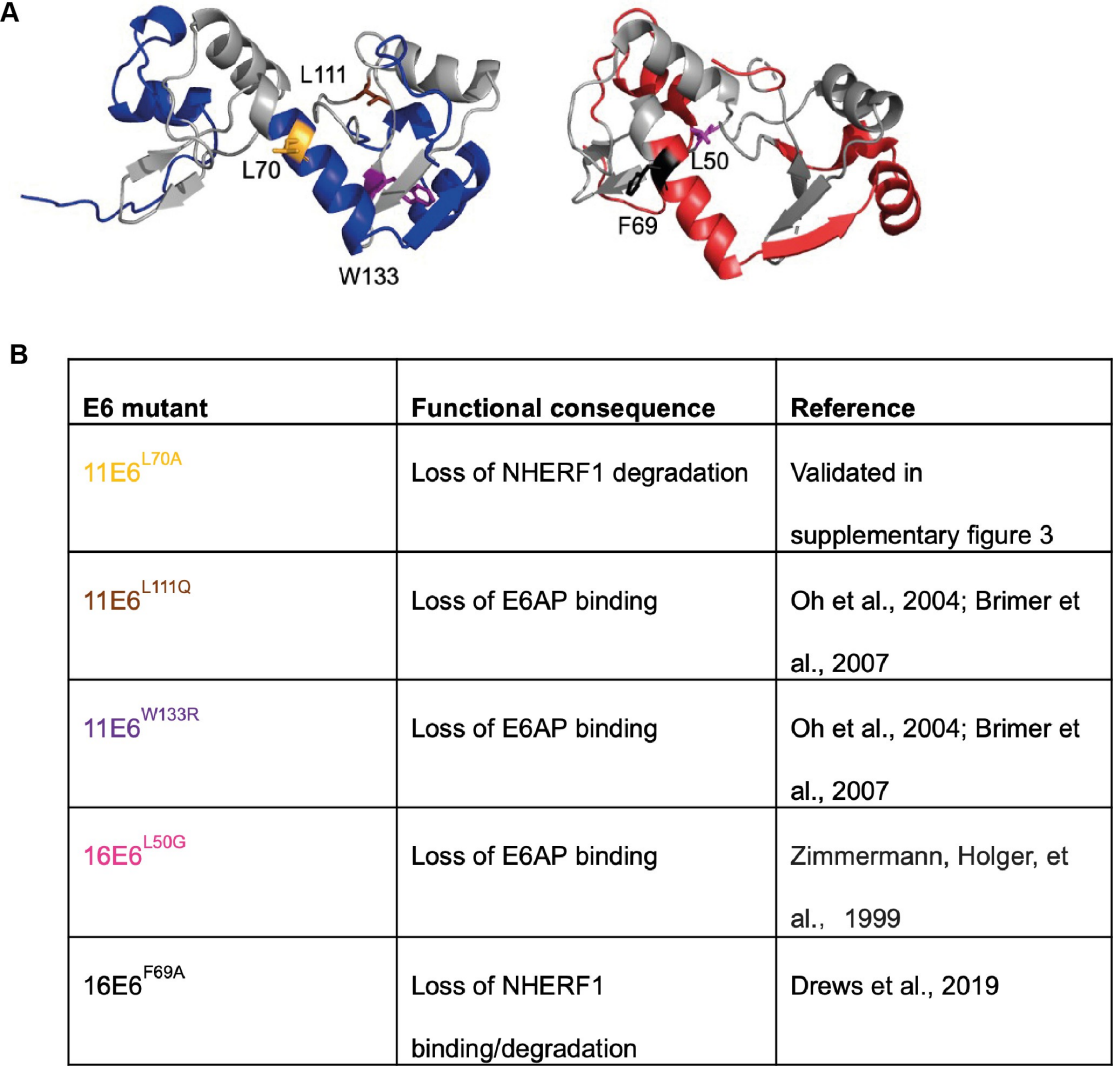
Summary of E6 mutants used in this study. (A) Structure of the ternary complex of 11E6 (predicted by Alphafold, [47] and 16E6 [48] with labelled point mutations. 11E6 (blue) with L70, L111 and W133 labelled. 16E6 (red) with F69 and L50 labelled. Zinc finger domains are displayed in grey. (B) Table that describes E6 mutants utilised in this work and associated functional defects.

**Fig 3:**
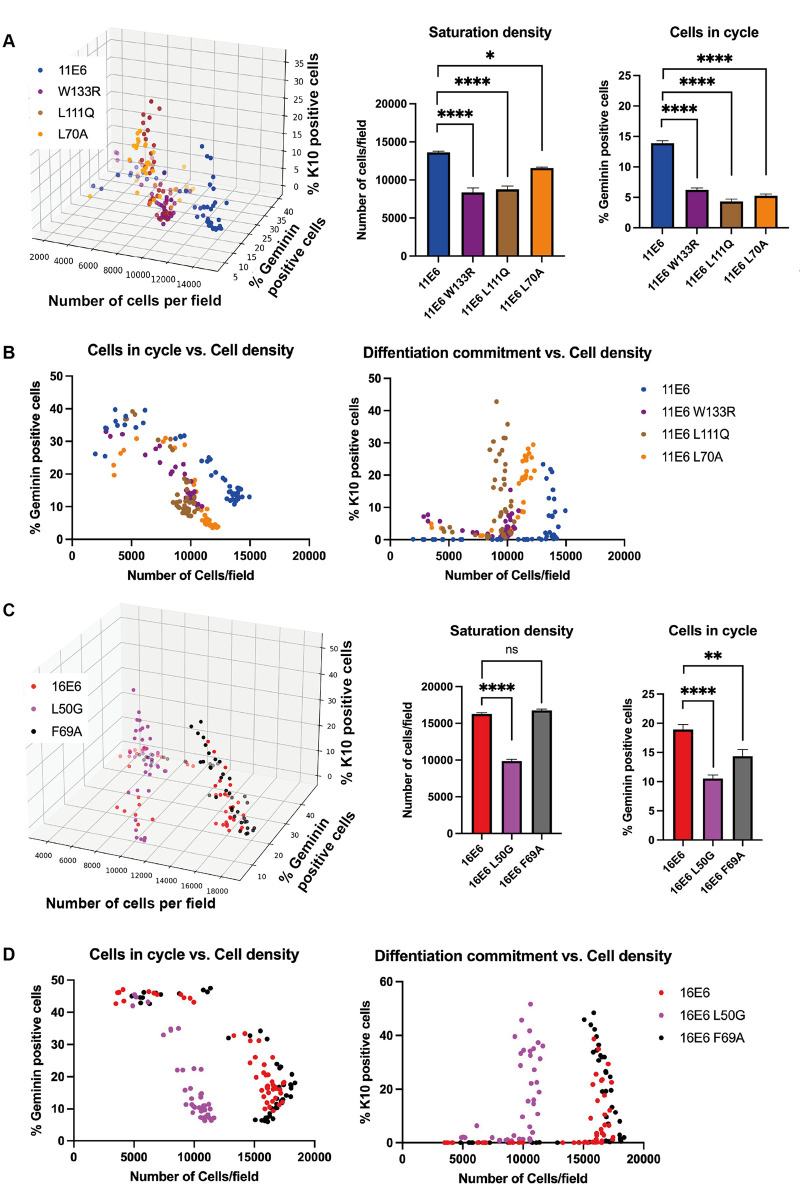
Role of E6AP and NHERF1 in E6-regulated keratinocyte phenotypes. (A-B) 11E6 requires E6AP binding or NHERF1 binding to modulate cells in cycle (% geminin-positive cells), differentiation commitment (% K10-positive cells), and saturation density (number of cells per field, each field = 3.78 mm^2^) of NIKS cells. (C-D) 16E6 requires E6AP but not NHERF1 binding to modulate cell cycle entry, differentiation commitment, and the saturation density of NIKS cells. Column graphs compare the saturation densities and % cells in cycle at saturation densities across cell populations. Statistical significance was determined by Kruskal-Wallis tests with Dunn’s correction. **, *P* ≤0.01; ***+, *P* ≤0.001; ****, *P* ≤0.0001.

After demonstrating that the E6 modifies the proliferation-differentiation steady-state, we next explored the possible mode of action by focusing on known E6 target proteins. E6AP is a conserved binding partner for Alpha group E6 proteins, leading us to consider whether E6AP may play an important role in the process. In addition, NHERF1 was one of the first cellular proteins discovered that can be degraded by both high and low-risk E6 proteins[27,32]. NHERF1 interacts with a range of signalling proteins including YAP, PTEN and frizzled receptors [31,42,43]. Several previously described E6AP-binding deficient mutants [26,44,45], 11E6^W133R^, 11E6^L111Q^ and 16E6^L50G^, were constructed (Fig 2). NHERF1-binding deficient 16E6^F69A^ mutant was established based on the work of Drews *et al*., 2019 and a corresponding point mutation in 11E6 was also generated (11E6^L70A^). Both mutants were shown to be deficient for NHERF1 degradation (S3 Fig) and analysed along with the other E6 mutants in Fig 3. The expression levels of wild type E6 and E6 mutants were examined by qRT-PCR and western blot analysis (S4 Fig). Intriguingly, the ability of E6 to modulate cell cycle entry, differentiation commitment, and cell saturation density were compromised by the loss of E6AP binding. As shown in Fig 3A and 3B, cells expressing 11E6^W133R^ and 11E6^L111Q^ reached lower saturation densities in comparison to cells expressing wild type 11E6. The percentage of geminin-positive cells was also reduced at saturation, and cells began to differentiate at lower densities in these cell populations. Similarly, L70A mutation also abolished 11E6’s ability to regulating cell cycle entry, saturation density and differentiation commitment, suggesting an important role for NHERF1 degradation in 11E6 functions. As expected, the L50G mutation abolished 16E6’s homeostatic functions as well (Fig 3C and 3D). However, the 16E6^F69A^ mutant retained most of the phenotypes of wild type 16E6, suggesting a less important role for NHERF1 in 16E6’s activity. Because E6AP is a direct target of Alpha group E6 proteins, while NHERF1 is one of the secondary substrates of the 16E6-E6AP complex, it is possible that other identified targets of 16E6 such as DLG, scribble and other PDZ proteins contribute to this process [46].

### E6AP but not NHERF1 contributes to E6’s competitive advantage in the lower keratinocytes layer

**Fig 4:**
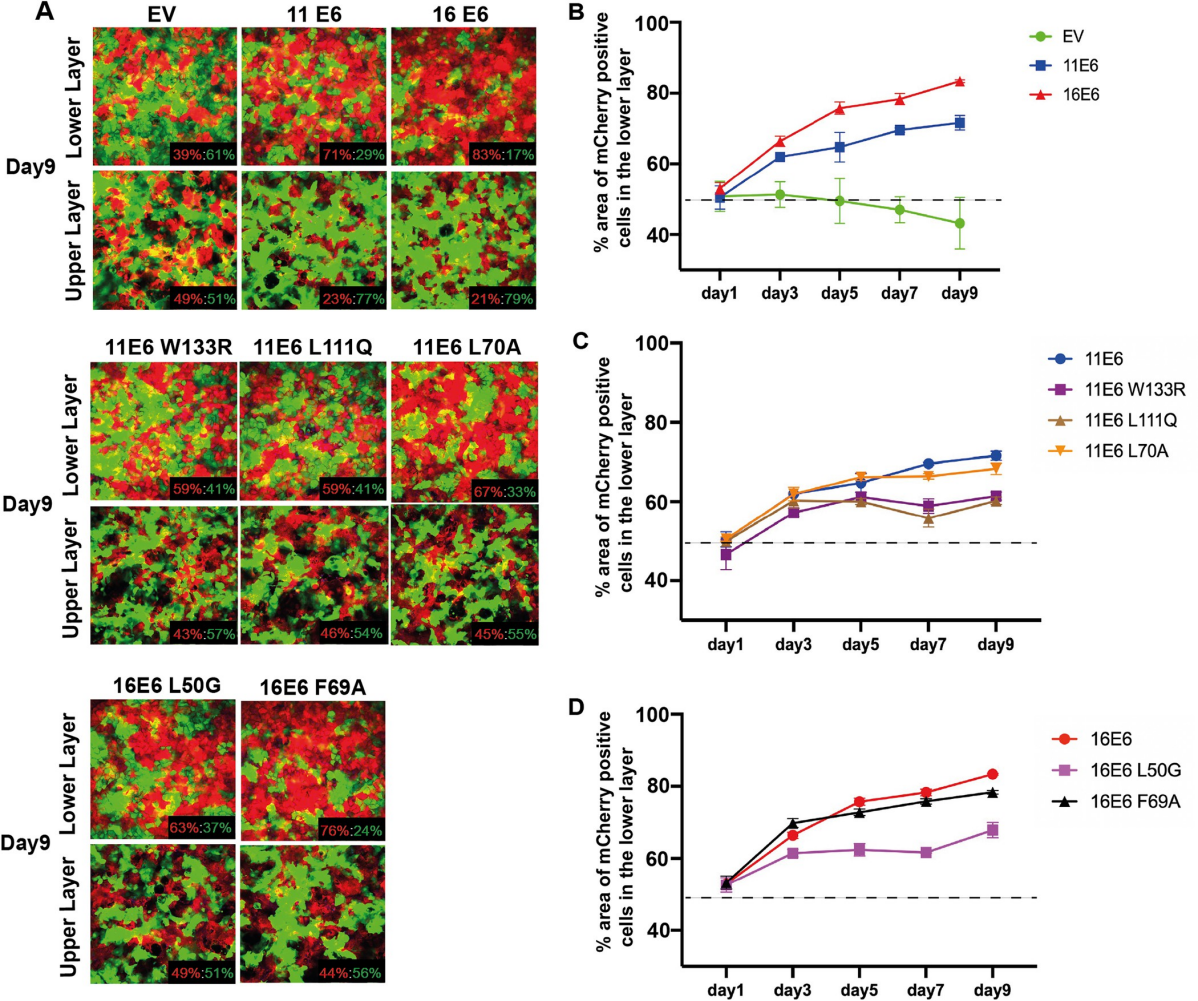
NIKS cells expressing E6 preferentially persist in the lower layer of cells in high-density competition assays. (A) NIKS^eGFP^-EV cells were seeded together with NIKS^mCherry^-EV or NIKS^mCherry^ cells expressing 11E6/11E6^W133R^/11E6^L111Q^/11E6^L70A^/16E6/16E6^L50G^/16E6^F69A^ at 50:50 ratio, and were cultured for nine days. The plates were then fixed by 4% PFA at day 1, 3, 5, 7 and 9, followed by staining with DAPI. The lower layer and upper layer of cells were scanned by Confocal microscopy. Representative images of the lower and upper layers of each group on day nine are shown. Ratio of area occupied by NIKS^mCherry^ to NIKS^eGFP^ is presented at the lower right corner of each image. Images were captured and quantified by Harmony high content imaging and analysis software (Perkinelmer). Original magnification: x20. (B-D) Graphs showing the % area of NIKS^mCherry^ in the lower layer during the competition assay. The area of NIKS^mCherry^ was quantified for each group of cell lines and data are means ± standard errors of three biological replicates. The dotted line marks 50% of the lower layer area of each field.

Given E6’s functions identified above, we then sought to explore the biological consequences of modifying these functions at the cell population level. Previously described cell-cell competition assays [38] enabled us to study not only the ‘enhanced fitness’ that E6 confers on keratinocytes in the lower (basal) epithelial layer, but also allowed us to assess effects on keratinocyte delamination. NIKS^mCherry^ cells expressing either E6^WT^ or E6 mutants, and NIKS^eGFP^-EV cells were seeded at 50:50 ratio to form a confluent monolayer on day one. Over a course of nine days, the cell populations were grown to allow the formation of at least two layers and fixed at each time point. Images of the lower and upper keratinocyte layers were captured by Z-stack confocal microscopy (Fig 4A). Day nine images are presented to show the most obvious effect. Although inhibitory effect on cell growth was observed for NIKS^mCherry^ cells (Fig 4B), the 11E6- and 16E6-expressing NIKS^mCherry^ cells gradually increased in proportion and outcompeted NIKS^eGFP^-EV cells, reaching 71.6% and 83.3% coverage respectively in the lower layer on day nine. This suggests that the NIKS^eGFP^-EV cells were ‘less-fit’ as they were displaced by NIKS^mCherry^-E6 cells from the lower layer and moved to the upper layer (Fig 4A). Furthermore, we found that the competitive advantage of 11E6 was compromised by E6AP binding deficiency. On day nine, both NIKS^mCherry^-11E6^W133R^ and NIKS^mCherry^-11E6^L111Q^ cells reached about 60% in the lower layer, and they also lost the ability to displace NIKS^eGFP^ cells into the upper layer (Fig 4A). Interestingly, the NHERF1-binding mutant 11E6^L70A^-expressing NIKS^mCherry^ cells behaved in a similar manner as NIKS^mCherry^-11E6^WT^, suggesting NHERF1 may not be involved in E6 function during cell competition. In parallel, we noticed that the competitive advantage of 16E6^L50G^ mutant-expressing NIKS^mCherry^ cells, which is deficient in E6AP association, was also significantly compromised in comparison to NIKS^mCherry^-16E6^WT^ and occupied approximately 67.9% in the lower layer. 16E6^F69A^ mutant-expressing NIKS^mCherry^ cells resembled the trend of NIKS^mCherry^-16E6^WT^ cells at all time points, reaching about 79% at day nine. This further indicates that NHERF1 may not play an important role in E6 regulation of cell delamination. This is also consistent with recently published results [49].

### Condyloma staining revealed several cellular targets regulated during low-risk HPV productive lifecycle

**Fig 5:**
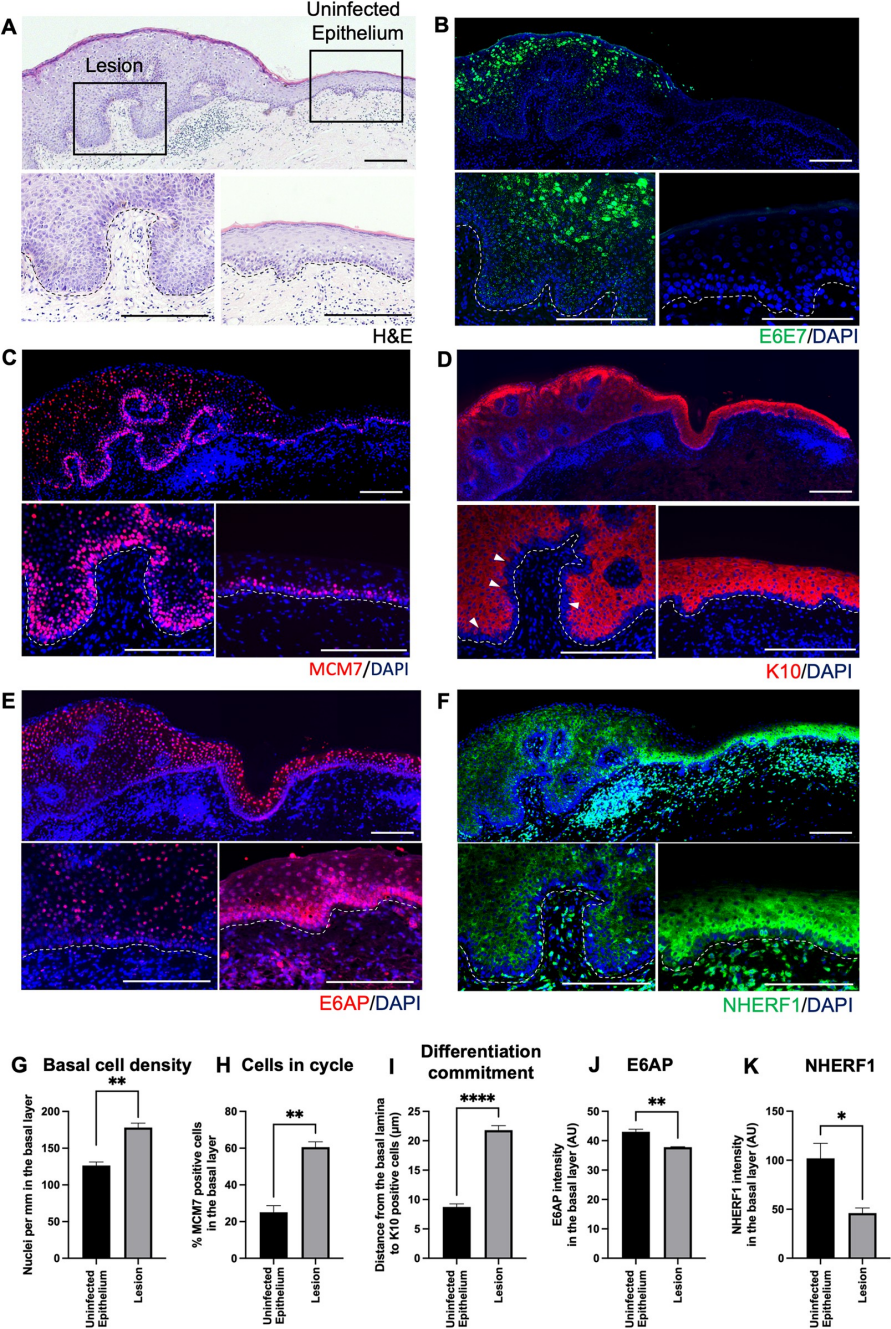
Localisation and expression pattern of E6/E7 and its targets during HPV11 productive lifecycle in condyloma acuminatum. (A) H&E staining of condyloma acuminatum biopsy with enlargement areas of lesion (left) and non-infected epithelium (right). (B-D) E6/E7 RNAScope, MCM7 and K10 immunofluorescence staining were carried out on adjacent sections. Nuclei were counterstained with DAPI (blue). Scale = 200 μm. Enlargement images are shown at the bottom, lower left is the lesion and lower right is the non-infected area. The dotted lines indicate the position of the basal layer. (E-F) E6AP and NHERF1 protein expression in infected (lower left) and non-infected (lower right) region of the biopsy. Scale = 200 μm. (G-I) The graphs show quantifications of basal cell density (nuclei per mm) by counting cells per 100 μm distance along the basal layers using Image J (G), % MCM positive cells in the basal layer (H), and distance from the basal lamina to the bottom of K10-positive cells (μm) (I). (J-K) show quantifications of E6AP and NHERF1 protein expression in the basal layer for HPV-infected and non-infected tissues. Integrated intensity (sum of the pixel intensity) of E6AP or NHERF1 stain in the basal layers of infected and non-infected tissues was measured using ImageJ. At least three different tissue samples and three representative images from each sample were included in the quantification. Graphs show mean values ± standard errors of amongst different condyloma biopsies. P values were calculated with student t tests. *, *P* ≤0.05; **, *P* ≤0.01; ****, *P* ≤0.0001.

Condyloma acuminatum is HPV-induced squamous epithelial proliferation in the anogenital region, caused by low-risk HPV types 6 and 11 [50]. Low-risk HPV infection-associated condyloma biopsy was stained with haemotoxylin & eosin (H&E) (Fig 5A). It comprised of both lesion and uninfected area, allowing us to make direct comparisons. The expression patterns of HPV 11E6E7 mRNA were identified in the epithelial basal layer of the lesion area using RNAScope (Figs 5B and S5). 11E6E7 mRNA expression was restrained in the basal layer and the lower layers of the lesion. The mRNA abundance increased significantly in the middle and upper layers. This is typical during low-risk HPV productive infection. In the same region, MCM7 and K10 staining were performed to provide indications for cell cycle and differentiation status (Fig 5C and 5D). Quantification of nuclei per mm shows an elevated cell density in the lesion when compared to uninfected epithelium (Fig 5G). The percentage of MCM7-positive cells was also significantly increased in the lesion, suggesting enhanced cell proliferation (Fig 5H). The increased distance from basal lamina to the cells starting to express K10 indicated a delay in the commitment to differentiation in the infected cells (Fig 5I), which is compatible with what was seen in the *in vitro* models (Figs 1 and 3 and S1 and S2). Additionally, we observed similar phenotypic changes in basal cell density, cells in cycle, and differentiation commitment in HPV16 associated low-grade squamous intraepithelial lesions (LSILs) (S6 Fig). The presence of E4 indicated HPV productive infections, and the increase in % MCM-positive cells was observed in the basal layer of the lesion (S6B Fig). Because there was little K10 expression in the cervical mucosal epithelium where *in vivo* HPV16 infection occurs, we used K13, which is the primary differentiation-dependent keratin expressed at this site (S6C and S6D Fig). A conspicuous delay in K13 expression was seen, and is marked by the white arrows, with changes quantified in S6E Fig.

To assess the clinical relevance of our cell culture observations, E6AP and NHERF1 staining was carried out on to adjacent sections of HPV11 condyloma biopsies. The localisation and expression patterns of these proteins were examined across infected and non-infected tissue areas, which allowed relative changes in abundance to be determined. To the best of our knowledge, there is no previous report describing the distribution of E6AP in stratified epithelial tissue or in HPV-infected tissue. Here we found that in non-infected epithelium adjacent to HPV-infected epithelium, there is heterogeneity in the patterns of E6AP expression amongst the cells of the basal layer with E6AP appearing in both the nucleus and cytoplasm (Figs 5E and S7). From the second layer and above, E6AP nuclear localization became progressively more evident. In the lesion area where papillae are apparent, there was significant reduction of nuclear E6AP in the upper layers. Although a heterogenous distribution of E6AP is apparent in the basal layer, the intensity of E6AP staining was decreased in comparison to the non-infected area (Fig 5J). Overall, there was a general reduction in E6AP protein abundance in the lesion. This agrees with previous *in vitro* findings that E6 can induce the auto-ubiquitination of E6AP and its degradation [51]. Further, our *in vitro* studies showed that NIKS cells stably expressing either 11E6 or 16E6 had lower E6AP protein levels compared to control cells (S7A Fig). In 3D organotypic rafts, reduction of E6AP was identified throughout the bottom and upper layers (S7B Fig). In addition, NHERF1 was mostly expressed from the second layer and into the upper layers in non-infected area, which displayed cytoplasmic and perinuclear patterns (Fig 5F). There were occasional basal cells that were found expressing NHERF1. However, NHERF1 levels were remarkably decreased in the lesion (Fig 5K), supporting previous literature that E6 degrades NHERF1 in various cell lines *in vitro*[27,32].

### E6 regulates YAP localization and phosphorylation level via E6AP and NHERF1

**Fig 6:**
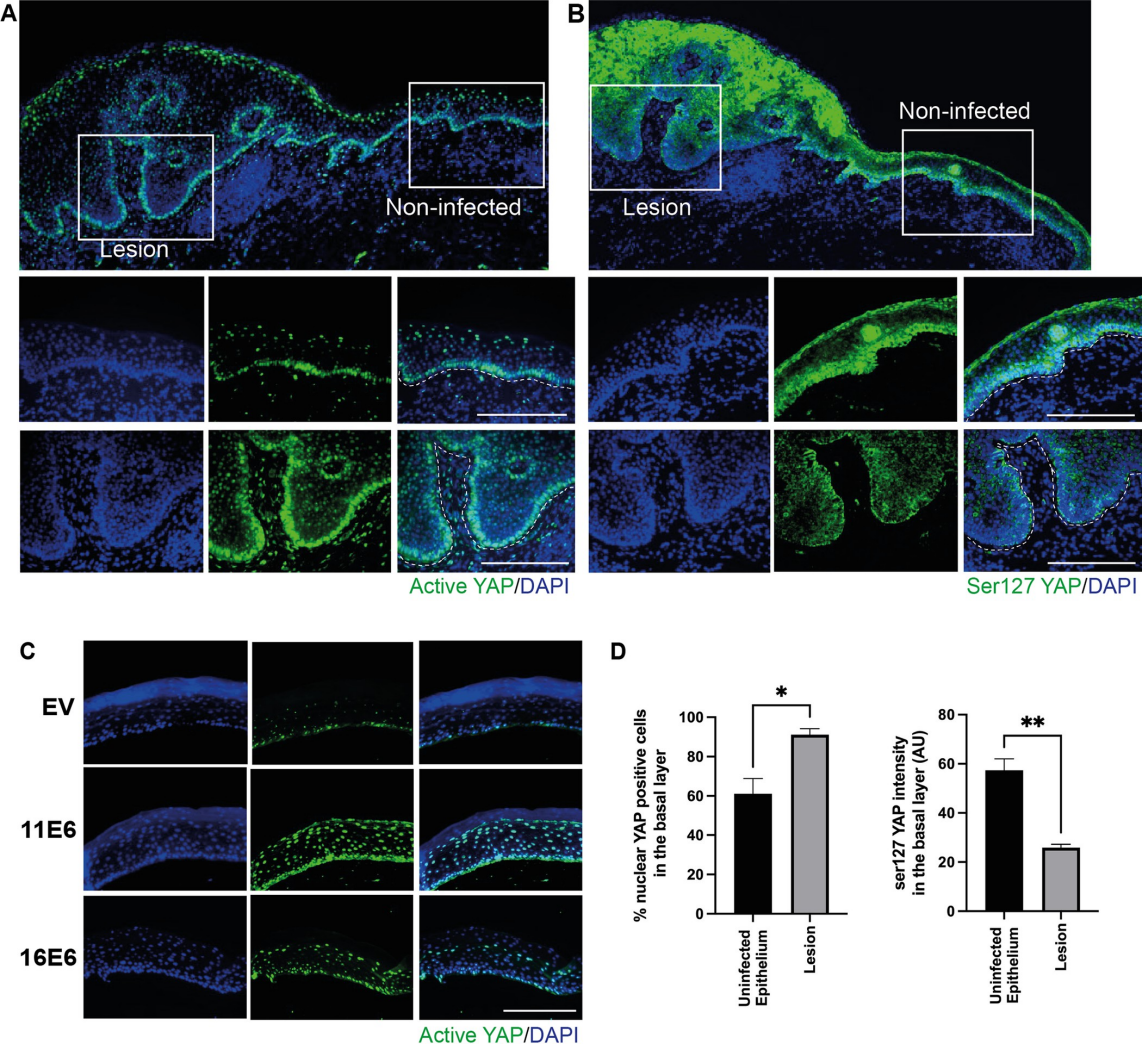
Both high and low-risk E6 promote YAP nuclear localisation in the basal layer. (A-B) Active YAP antibody (Abcam, ab205270) that recognises un-phosphorylated form of YAP and p-YAP (Ser127) antibody (Cell signalling, 4911) that only recognizes YAP phosphorylated at position Serine 127 were used for staining on condyloma biopsies. Nuclei were stained with DAPI. Specific areas of lesions and non-infected are shown as enlargement images at the bottom, lower left is the lesion and lower right is the non-infected epithelium. Scale = 200 μm. (C) Organotypic rafts of NIKS transduced with retroviral vectors encoding EV, 11E6 and 16E6 were established, sectioned and stained with active YAP antibody. Scale bar = 200 μm. (D) Quantifications of % cells with nuclear YAP and phosphorylated YAP (Ser127) levels in the basal layer of uninfected epithelium and low-risk HPV infected lesions. At least three different tissue samples were included in the quantification. Graphs show mean values ± standard errors of amongst different condyloma biopsies. P values were calculated with student t tests. *, *P* ≤0.05; **, *P* ≤0.01.

YAP has been identified as a critical modulator involved in sensing cell density and the regulation of cell proliferation, and has been shown to have important functional roles in keratinocyte homeostasis [22,52,53]. YAP localization is mainly regulated through phosphorylation by LATS1/2 [54]. At high cell density, a major phosphorylation of YAP occurs on the position Serine 127 (Ser127), leading to YAP sequestration in the cytoplasm [55]. At low cell density, YAP is not phosphorylated and enters the nucleus to activate downstream genes [56]. Past work has demonstrated that high-risk HPV E6 proteins regulate the Hippo signalling cascade during the progression to cervical cancer [20]. However, we suspected that both low-risk and high-risk E6 proteins manipulate YAP activity in low-grade lesions to adjust homeostasis. Therefore, we examined the localisation and abundance of YAP in the condyloma tissue by using antibody that specifically recognises the non-phosphorylated (active) form of YAP1 (Fig 6A). In non-infected epithelium, YAP is predominately nuclear when it is present in the basal cells. This is consistent with the observations from previous reports [52,57,58]. Nuclear YAP gradually decreased in the suprabasal and upper layers and became more prominent in the granular or cornified layer. By comparison, the number of cells with prominent level of YAP increased in the basal layer in the infected area, suggesting a subtle modulation of homeostasis towards proliferation. Ser127 YAP was found significantly decreased in the basal and suprabasal layers, whereas relatively high-level expression of Ser127 YAP is observed in non-infected tissue (Fig 6B). Quantification of nuclear YAP and Ser127 YAP expression in the lesion versus non-infected epithelial basal layers was performed using at least three different tissue samples (Figs 6D and S8). The significant increase of % nuclear YAP and reduction of Ser127 YAP expression in the basal layer indicated that YAP nuclear-cytoplasmic shift was modulated during low-risk HPV infection.

**Fig 7:**
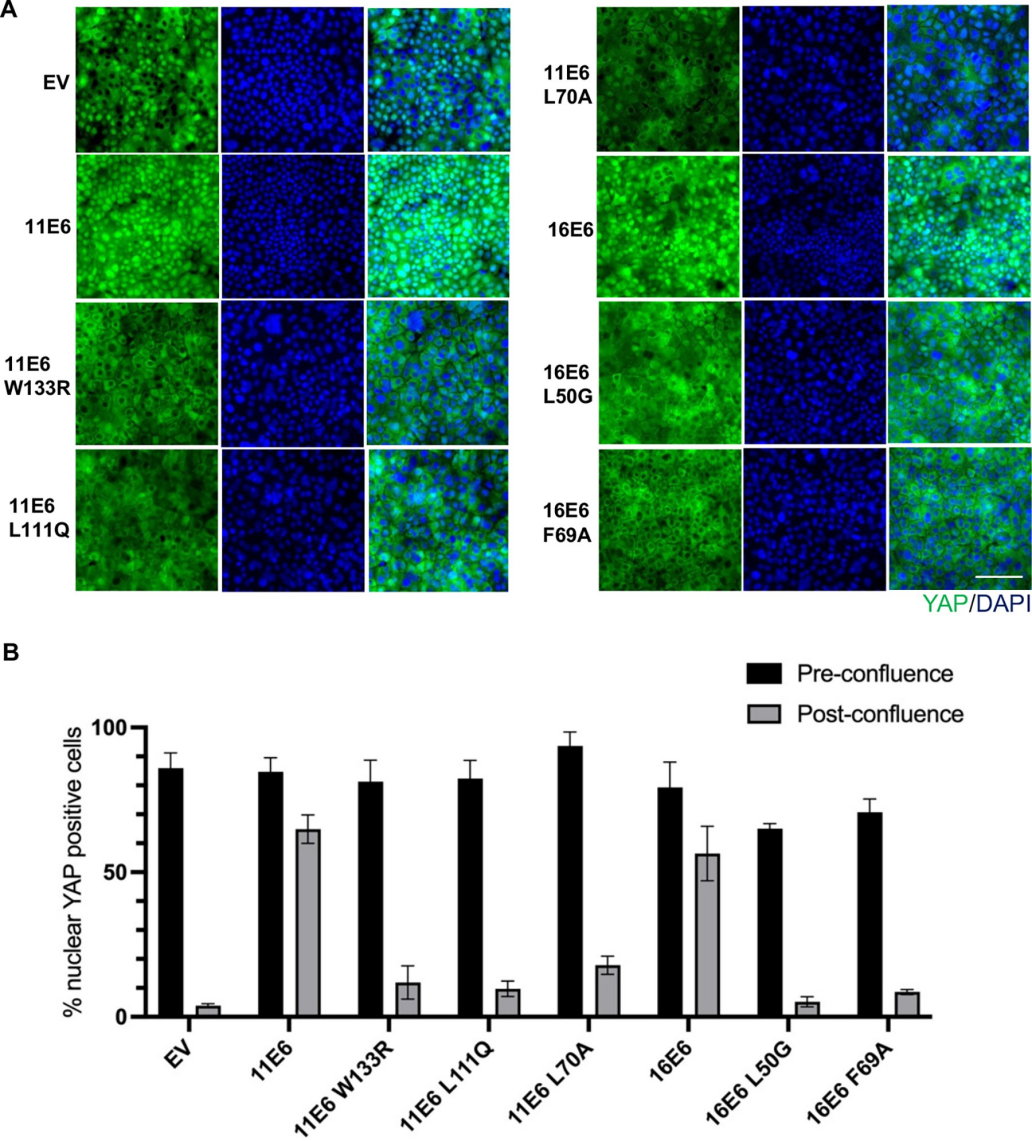
E6 requires E6AP and NHERF1 to enhance YAP nuclear localisation. (A) NIKS transduced with either EV or retroviral vectors encoding 11E6/16E6/11E6^W133R^/11E6^L111Q^/11E6^L70A^/16E6^L50G^/ 16E6^F69A^ were seeded at 8x10^5^ cells per well in six-well plate. After 72 hours, cells were fixed and stained with active YAP antibody and DAPI. Scale = 200 μm. (B) Quantification of % nuclear YAP positive cells at pre- and post-confluence in NIKS cells expressing E6 or E6 mutants. Data are means ± standard errors of three biological replicates.

In parallel, NIKS transduced with retroviral expression vectors encoding either 11E6 or 16E6 were used to grow organotypic cultures, which displayed higher level of nuclear YAP in the basal layer of the rafts relative to parental NIKS raft (Fig 6C), supporting our observations in condyloma tissues. To study how E6 overcomes the impact of contact inhibition through YAP activation, we seeded FUCCI NIKS cells expressing E6 or E6 mutants at post-confluence and stained with active YAP. 11E6 and 16E6 both led to enhanced nuclear YAP relative to NIKS-EV, whereas the E6 mutants 11E6^W133R^, 11E6^L111Q^, 11E6^L70A^, 16E6^L50G^ and 16E6^F69A^ lost the ability to retain YAP in the nucleus (Fig 7). This suggests that E6AP and NHERF1 are critical in regulating YAP nuclear localisation. Knockdown of E6AP disrupted the effect of E6 on YAP relocalisation (S9 Fig). While total YAP maintained at similar levels, E6-expressing NIKS cells had elevated active YAP and reduced Ser127 YAP levels at post-confluence, whereas the mutant cell lines had similar levels of Ser127 YAP as NIKS-EV (S10 Fig). This further implies that E6 requires NHERF1 and E6AP binding to promote YAP nuclear localisation at post-confluence, and this leads to the reduction of Ser127 phosphorylated YAP in cytoplasm.

### E6AP is important for cell differentiation

**Fig 8:**
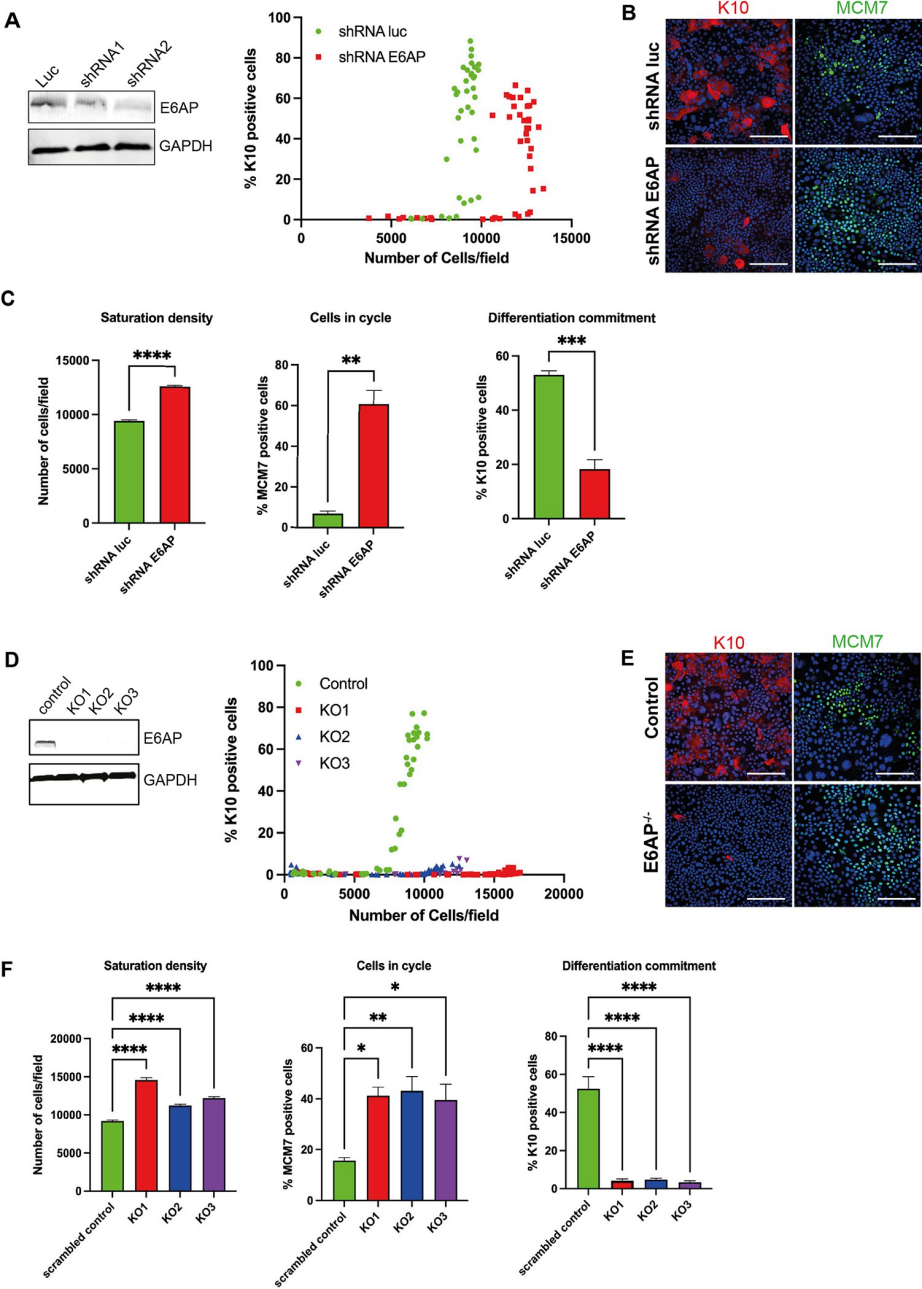
E6AP is a critical regulator of keratinocyte differentiation. (A) NIKS cells were retrovirally transduced with plasmids expressing shRNA targeting E6AP or luciferase. Cells were then selected with puromycin and validated with western blot. Cells were plated at different densities and after 72 hours, cells were stained with K10 and DAPI. % K10-positive cells was plotted against the number of cells per field. (B-C) NIKS-shRNA-luc and NIKS-shRNA-E6AP cells at post-confluence were stained with K10 (red) and MCM7 (green). Saturation densities, cells in cycle and differentiation commitment were measured for at least three representative images, mean values ± standard errors were calculated, and student t-tests were performed. **, *P* ≤0.01; ***, *P* ≤0.001; ****, *P* ≤0.0001; (D) NIKS E6AP^-/-^ and control cell lines were generated by transfecting cells with px459 plasmid expressing E6AP gRNA or a scrambled control. Puromycin selection and single cell selection were carried out to obtain three independent knockout clones. Western blot indicates the loss of E6AP in NIKS^E6AP-/-^ cells. Control and NIKS E6AP^-/-^ cells were plated at different densities and fixed after 72 hours. Cells were then stained with K10 and DAPI. % K10-positive cells was plotted against the number of cells per field. (E-F) Control and NIKS^E6AP-/-^ cells at post-confluence were stained with K10 (red) and MCM7 (green). Saturation density, cells in cycle, and differentiation commitment were measured for at least three representative images, mean values ± standard errors were calculated, and student t-tests were performed. *, *P* ≤0.05; **, *P* ≤0.01; ****, *P* ≤0.0001.

Based on the results shown above, our work demonstrates that E6AP plays an important role in E6-regulated homeostatic phenotypes in keratinocytes. Accumulating evidence shows that Alpha group E6 binding to E6AP leads to the activation of its ubiquitin ligase activity and self-degradation[44,51]. Together with our results, it prompts the hypothesis that E6 may regulate the natural cellular targets of E6AP by directly targeting E6AP for degradation. Thus, we transduced NIKS cells with shRNA oligonucleotides targeting E6AP. The knockdown effect was validated with western blot (Fig 8A). NIKS-shRNA-luciferase (control) and NIKS-shRNA-E6AP cells were plated at cell densities ranging from pre-confluence to post-confluence. After 72 hours, cells were fixed and stained with K10. NIKS-shRNA-E6AP cells displayed higher saturation density (13,000 cells/field) and delay of K10 expression in comparison to the control NIKS (Fig 8A). At the same cell density, MCM-positive cells were increased, whereas K10-positive cells were significantly reduced when E6AP was knocked down (Fig 8B and 8C). This provides evidence that E6AP contributes to the proliferation-differentiation switch in keratinocytes. Further, NIKS-shRNA-E6AP organotypic rafts were established, which allowed us to examine K10 expression in different layers. We found a slight delay of K10 expression in the second layer of NIKS-shRNA-E6AP raft in comparison with the control raft (S11 Fig).

In parallel, NIKS cell lines with E6AP knocked out by CRISPR-Cas9 were established. Three clonal E6AP^-/-^ NIKS cell lines with genome edited by plasmid expressing E6AP-gRNA were selected. Sequencing of the genomic region targeted by the gRNA confirmed frameshift mutations had been introduced into each allele and no wild-type (WT) allele remained. Consistent with this, immunoblotting showed loss of E6AP expression (Fig 8D). The E6AP^-/-^ NIKS cell lines were then plated in cell densities ranging from pre-confluence to post-confluence. After 72 hours, cells were fixed and stained with K10 (Fig 8E). Three E6AP^-/-^ NIKS cell lines all reached higher saturation densities, from 12,000 cells/field to 15,000 cells/field in comparison to the control cell line (8000 cells/field) (Fig 8F). K10 expression was remarkably reduced in E6AP^-/-^ NIKS cells after saturation density was reached. When comparing at similar cell densities, K10 expression was significantly lower and % MCM-positive cells were increased (Fig 8E and 8F). These results support our observations in NIKS-shRNA-E6AP cells and demonstrate a potential role for E6AP in modulating epithelial homeostasis. Next, we transduced 11E6 or 16E6 into E6AP^-/-^ NIKS cells and examined K10 expression at high cell density. In contrast to E6AP^-/-^ cells, % K10 positive cells further declined upon E6 expression, suggesting E6AP-independent functions of E6 (S12 Fig) must also contribute to the regulation of the proliferation-differentiation switch.

### E6 requires E6AP depletion to impair differentiation gene expression and activate YAP target genes in keratinocytes

**Fig 9:**
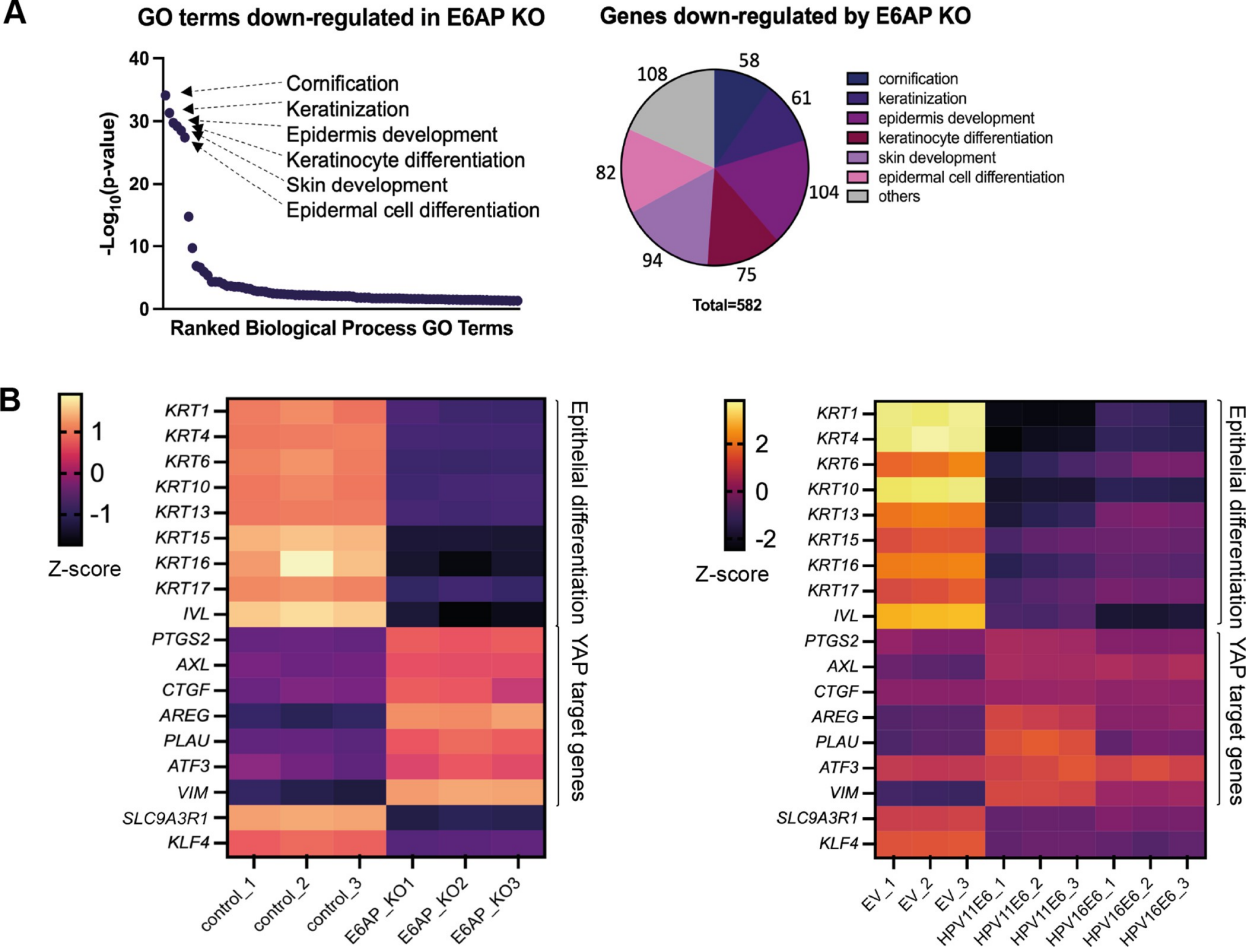
Similar transcriptional profiles were identified between E6AP^-/-^ and E6-expressing cells. Total RNA was extracted from NIKS control, NIKS E6AP^-/-^ and NIKS transduced with EV, plasmids expressing 11E6 or 16E6 when the cells reached post-confluence (cell density = 95,000 cells/cm^2^). PolyA selected RNA levels were measured by RNA sequencing, followed by differential gene expression analysis. (A) Gene ontology (GO) enrichment analysis of genes down-regulated in NIKS E6AP^-/-^ compared with NIKS-control. Pie chart displays the fraction of genes down-regulated in the absence E6AP that fall into enriched GO terms. (B) Heatmaps displaying mRNA expression differences across NIKS E6AP^-/-^ and NIKS-E6 samples in comparison to the control groups. Three biological repeats were included in the analysis. Selected epithelial differentiation genes and YAP-responsive genes that were significantly differentially expressed are shown in the heatmap. The legend shows the range of Log_2_ (FPKM+1) values of genes that are homogenised across the row (Z-score). The complete set of heatmaps is shown in S1 and S2 files.

**Fig 10:**
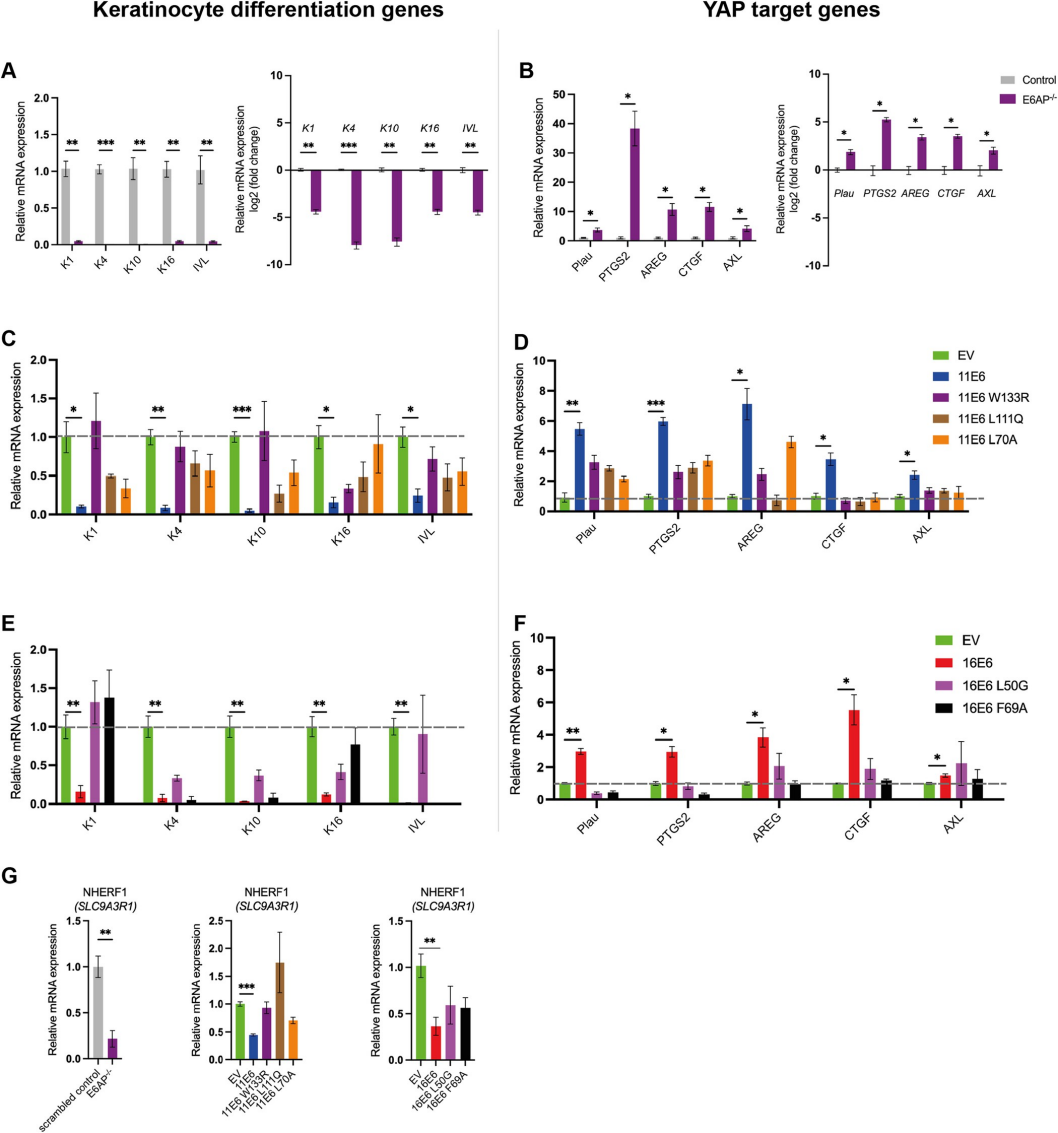
Both E6AP^-/-^ and E6 expression led to downregulation of keratinocyte differentiation genes and activation of YAP target genes. (A-B) qRT-PCR was used to measure the expression of the differentiation markers and YAP target genes relative to GAPDH in E6AP^-/-^ and control samples. Graphs represent the fold change in gene expression normalised by the control group. Graphs with Log2 (fold change) were shown on the right for better illustration. (C-F) The same set of genes were measured relative to GAPDH for the 11E6 and 16E6 groups of samples using qRT-PCR. The dotted line marks gene expression relative to the EV control. (G) NHERF1 (*SLC9A3R1*) expression was measured in all samples. All column graphs show mean +-standard errors of three independent experiments. Multiple t tests were performed to show statistical significance. *P < 0.05; **P < 0.01 ***P < 0.001.

To establish which cellular pathways are affected following E6AP loss in keratinocytes under condition which differentiation would normally be triggered, total RNA was isolated from three independent samples of NIKS-control and NIKS E6AP^-/-^ cells that grew until post-confluence. With the standard mRNA read depth of around 20 million reads/sample, 3824 genes were differentially expressed with fold-change > = 2 and adjusted P< = 0.05 in E6AP^-/-^ cells (S13 Fig). Of these, 1664 genes were down-regulated and 2160 genes were upregulated in the absence of E6AP. In the gene enrichment analysis, more than half of the down-regulated genes were in keratinocyte differentiation-related GO categories (Fig 9A and 9B). These included cornification, keratinisation, epidermis development, keratinocyte differentiation, skin development and epidermal cell differentiation. We selected a subset of markers of keratinocyte differentiation, including as keratin 1, keratin 4, keratin 10, keratin16 and involucrin (IVL) for validation by qRT-PCR. These genes were all significantly downregulated in E6AP^-/-^ cells (Fig 10A). Keratin 1, 4, 10, and 16 are associated with the suprabasal layers of differentiating keratinocytes [59,60]. IVL is also a marker for keratinocyte differentiation commitment which is expressed at high levels in the suprabasal layers of the epidermis before cornification occurs [61].

Additionally, relevant GO categories upregulated by E6AP^-/-^ included regulation of signalling receptor activity, extracellular matrix organisation and cell-cell adhesion (S13C Fig). Among these GO terms, we found a subset of YAP target genes were significantly upregulated (Fig 9B). qPCR was then performed to quantitate the relative abundance of YAP downstream gene expression (Fig 10). Consistent with the RNA sequencing outcome, well-characterised YAP target genes *AREG*, *PLAU*, *PTGS2*, *AXL* and *CTGF* that were described in previous literature [22,62–64], were expressed at 2- to 40-fold higher levels when E6AP was depleted (Fig 10B). All these genes have indicated functions in driving cell proliferation.

Given our finding that E6 requires E6AP binding to promote YAP nuclear localisation (Figs 6 and 7), we hypothesised that E6 inhibits E6AP function to activate specific YAP target genes. Total RNA was extracted from NIKS-control and NIKS cells stably expressing either 11E6 or 16E6 that grew until post-confluence to trigger differentiation, and sent for RNA sequencing. For the GO enrichment analysis, both 11E6 and 16E6 downregulated differentiation-related processes including cornification, keratinisation, keratinocyte differentiation etc (S13C Fig). At the same time, cell cycle-related processes including G2/M phase transition, G1/S phase transition and cell division were upregulated. These results correlate to the increased proliferation and delayed differentiation of NIKS cells under the expression of E6 presented above (Fig 1). A subset of YAP target genes was also found to be activated in E6-expressing NIKS cells (Fig 9B). To confirm that E6AP degradation is required for YAP target gene activation, NIKS cells expressing E6 or the mutants were seeded at high cell density and the mRNA abundance was quantified by RT-qPCR. Indeed, 11E6^W133R^, 11E6^L111Q^ and 16E6^L50G^ which cannot induce E6AP degradation had reduced ability to upregulate YAP-responsive genes (Fig 10D and 10F). Interestingly, RNA sequencing and qPCR results suggest that NHERF1 gene (*SLC9A3R1*) downregulation is found in E6-expressing and E6AP^-/-^ NIKS cells (Fig 10G). Thus, NHERF1 is not only degraded by E6-E6AP complex but also downregulated by E6 at transcriptional level. It is possible that NHERF1 reduction directly controls YAP transcriptional activity, because E6 mutants 11E6^L70A^ and 16E6^F69A^ cannot upregulate YAP target genes.

## Discussion

Human papillomaviruses establish chronic lesions in the epithelium[4,65,66]. During evolution, HPVs have become adapted to distinct epithelial niches by manipulating molecular processes, and as a result of this, have developed distinct tissue tropisms[11,67]. The Alpha genus E6 proteins for example, preferentially bind to E6AP, whereas the E6 proteins from the other genera confers stronger interaction with MAML [25,68,69]. In both instances, it appears that this leads to the inhibition of Notch signalling and delay of the terminal differentiation program [68,70,71]. Recent published work on MmuPV and the high-risk E6 proteins have indicated that E6 rather than E7 has a dominant role in driving cell competition and promoting basal cell retention [38,49], and that the low-risk E6 protein is a key driver of keratinocyte proliferation, with a significant role in delaying keratinocyte committment to differentiation[13,14]. Accumulating evidence suggests, that the E6 proteins have conserved functions in modulating the balance between cell cycle progression and differentiation, which are crucial for HPV-infected lesion expansion and persistence. Our work, which dissects the shared functions of the high- and low-risk HPV E6 proteins, concludes that they target similar homeostatic processes, and that despite differences in binding partners, that in both cases, E6AP serves an important role.

For many of our experiments, we focused on the expression of the E6 protein rather than expression from the whole genome, partly because of the suggested role of E6 in modulating homeostasis, but also because we wished to understand its role in the isolation of other viral gene products. The relevance of the effects seen with E6 alone in of tissue culture models, is however apparent from the analysis of *in vivo* -derived clinical material infected with HPV11, and also with HPV16, as similar changes in basal cell density, cell cycle activity and differentiation were also evident in patient-derived clinical material (S2 and S5 Figs). Although it is likely that other viral proteins play a role in homeostasis regulation, comparison of the E6-mediated phenotype with those seen *in vivo* in tissue, and in in HPV16 genome rafts, suggests that E6 is an important player. Although HPV11 causes persistent benign lesions *in vivo*, the HPV11 genome cannot be maintained over repeated passage in tissue culture, a limitation which has compromised our understanding of the low-risk HPV types. It is likely that additional high-risk HPV functions involved in driving cell cycle entry have contributed to our ability to study the high-risk viruses. Despite these limitations, previous studies in normal human keratinocytes have suggested that episomal maintenance is further compromised in the context of 11E6^W133R^, 11E6^L111Q^ and 16E6^L50G^ mutnts [45,72,73]. It appears therefore, that E6AP-binding deficient E6 proteins may lose the ability to support viral genome maintenance in addition to affecting normal homeostasis functions, and indeed, our preliminary results show that even the HPV16 genome cannot be maintained in E6AP^-/-^ NIKS cells. In our hands, the HPV11 genome is lost rapidly in both E6AP^-/-^ cells and control cells (S13 Fig). Although these issues limit the investigating E6 functions in whole genome background, it is apparent that E6 modulates cell phenotype in order to increase the cell density at which proliferation gives way to differentiation.

From our results, it appears that both high and low-risk E6 expression increases the proportion of cycling cells at both pre-confluence and post-confluence. Because E6 has a prominent role in overcoming normal keratinocyte contact inhibition[13,74,75], cells expressing E6 typically reached a higher saturation density than the control cells. Nevertheless, 16E6-expressing cells always reach higher saturation density than 11E6-expressing cells. Similarly, NIKS transduced with either 11 or 16E6 typically start to trigger K10 expression at higher cell density than NIKS-EV, with 11E6 causing and intermediate phenotype between the two. In cell-cell competition assays, we also examined the effect of E6 expression on the progression of cells from the first layer to the second layer, which we suggest is analogous to progression from basal to parabasal cell layers. Our results are in line with current thinking, that E6 expression retains keratinocytes in the basal layer and delays delamination, while the wild-type NIKS cells were displaced and entered the second layer [38,49]. Consistent observations in primary human keratinocytes have been suggested by other groups, or shown by our results (S1 Fig). Although 11E6 appeared to have an intermediate phenotype, both E6 proteins confer a basic set of functions to modulate cellular phenotypes involved in homeostasis. In each case, E6AP plays an important role. The more subtle cellular phenotypes caused by 11E6 expression compared to 16E6 could be partially due to their different modes of interaction with E6AP. It has been suggested that 11E6 and 16E6 bind to various auxiliary regions on E6AP, which leads to distinct substrate degradation[76]. For example, 11E6 cannot cause p53 degradation but degrades NHERF1 in a similar manner to 16E6. Previous *in vitro* binding experiments and co-immunoprecipitation studies also showed that the interaction between 11E6 and E6AP is weaker than 16E6-E6AP binding [73,77]. Additional roles of 16E6 may also contribute to the phenotypes described. The ability of 16E6 to degrade PDZ proteins is E6AP-independent and may contribute additional to the modulation of homeostasis [78,79]. Transcriptional regulation of 11E6 or 16E6 through the interaction with p300 has been shown to affect downstream cellular targets such as p53 [80,81], which is expected to confer additional functions on E6. In order to demonstrate the physiological relevance of our *in vitro* assays, we used a number of additional systems, including primary human keratinocytes (NHEK) in addition to NIKS, the use of organotypic raft culture, and most importantly, the evaluation of *in vivo* patient-derived clinical material (Figs 5 and S6), which revealed the extent of basal cell density and cell cycle changes typically seen during benign productive infections.

Currently, Hippo signalling has emerged as one of the key pathways being altered frequently in HPV-related cancers [18]. It has been suggested that high-risk E6 requires the PDZ motif to promote YAP nuclear localisation in serum-starved keratinocytes[21]. High-risk E6 was also shown to drive cervical cancer cell proliferation by maintaining high levels of YAP in cells, with YAP expression being correlated with cervical cancer progression [20]. More recently, high-risk HPV E7 was proposed to activate YAP1 in basal keratinocytes by degrading PTPN14, which contributes to papillomavirus persistence and carcinogenesis [82,83]. Despite its critical involvement in carcinogenesis, YAP is also required for normal skin homeostasis [31,84]. The proliferation of basal layer cells was significantly reduced in YAP/TAZ double knockout mouse skin [52]. Our findings revealed that YAP1 nuclear translocation can be promoted by low-risk 11E6 as well, and this is achieved through E6AP binding, suggesting YAP1 activation is involved during both low-risk and high-risk HPV infections (Fig 7). The highly conserved feature of E6 binding to E6AP indicates that YAP1 activation and maintenance of basal cell state is likely shared among diverse Alpha genus E6 proteins. Interestingly, RNA sequencing performed using human basal layer keratinocytes indicated that YAP transcriptional regulation is occurs primarily in the basal cell population [52]. This correlates with our observation that active YAP expression is identified in the basal layer of condyloma sections, and is upregulated during ongoing low-risk HPV infection (Fig 6). Furthermore, RNA sequencing and qPCR validation demonstrated that YAP downstream genes were activated in the presence of either 11E6 or 16E6, and that loss of E6AP binding abolished E6’s ability to stimulate the expression of Yap-responsive genes (Fig 9). Our results are broadly consistent with previous findings which showed that that *PLAU* and *PTGS2* are upregulated *in vivo* by constitutive YAP activity in mouse skin, as well as in HaCat keratinocytes grown *in vitro* [22]. Thus, both 11 and 16E6 proteins drive keratinocyte proliferation by activating YAP downstream genes. Interestingly, it was recently shown that YAP/TAZ can also regulate genes involved in keratinocyte differentiation in the basal layer of organotypic raft culture [82]. YAP activation leads to a crosstalk with Notch signalling pathways through the upregulation of the Notch ligands DLL1, JAG2 and DLL3, leading eventually to cell-autonomous cis-inhibition of Notch [24]. It is thought that this allows epidermal progenitors to be maintained in an undifferentiated state, which fits well with a. role for E6 in maintaining a stem-like/progenitor cellular phenotypes during infection. Thus, it appears that the local microenvironment is dynamically regulated by E6, in order to adjust the balance between cellular self-renewal differentiation favours persistence of the infected basal cell.

Alpha genus E6 proteins deplete E6AP to different extents by inducing the self-ubiquitination and degradation of E6AP[44,51]. Importantly, our clinical observations show that in the basal layer where E6/E7 viral genes are expressed, a reduction of cytoplasmic E6AP was observed in comparison with the uninfected epithelium. In the upper layers, although E6AP accumulates in the nucleus, its abundance is noticeably less prominent in the presence of amplified E6/E7 expression (Fig 5). This is the first description of E6AP protein expression pattern in human tissue, which agrees well with our *in vitro* work that NIKS cells transduced with E6 result in a decreased in endogenous E6AP protein level (S7 Fig). However, the consequence of E6AP degradation in the context of HPV life cycle and epithelium homeostasis has not been fully explored. Our results show that loss of E6AP binding diminishes a major component of both high and low-risk E6 function in driving cell cycle entry, delaying differentiation, overcoming contact inhibition and basal cell retention. Because high-risk E6 targets p53 to delay keratinocyte differentiation, the low-risk E6 proteins, which cannot direct p53 degradation, may target E6AP directly and affect its inherent regulatory functions. Indeed, our shRNA-E6AP and E6AP^-/-^ cell lines both demonstrated that E6AP is required for the normal keratinocyte differentiation program, and that its depletion leads to fewer cells committing to differentiation, and instead remaining in cycle. Additionally, past literature suggests that E6AP has an impact on cell cycle control and proliferation [28,29,85]. E6AP is known to possess two independent roles: a transcriptional coactivator and an E3 ubiquitin ligase [86]. Apart from Hippo signalling, our RNA sequencing data provides a number of cellular pathways that could be regulated by E6AP transcriptionally, including the Wnt and PI3K-Akt signalling pathways. Both pathways have been implicated in the regulation of cellular growth [50,87,88]. Data from E6AP deficient mice suggests that p53 levels might be affected by the absence of E6AP [86], with deregulation of p53 levels in epithelium known to affect keratinocyte differentiation [15]. Other substrates of E6AP such as the polycomb protein Ring1B and PML (promyelocytic leukemia) tumour suppressor were also reported previously to modulate proliferation-differentiation balance [89–92].

It is possible that E6 regulates the levels of natural cellular targets of E6AP by inducing its degradation. On the other hand, E6 modifies E6AP substrate specificity to degrade other cellular proteins, which may also contribute to the phenotypes we observed, with E6AP and NHERF1 both depleted in cells expressing either 11E6 or 16E6. Interestingly, NHERF1 expression levels were found to decline in the absence of E6AP (Fig 9). RNA-seq results showed the resemblance of E6-expressing to E6AP^-/-^ keratinocytes, and that both displayed lower expression of keratinocyte differentiation-related genes and an activation of YAP downstream genes. NHERF1 has been shown to directly interact with YAP and its depletion leads to YAP translocation to the nucleus [31]. Both E6 expression or E6AP knockout resulted in reduced NHERF1 mRNA expression in our RNA sequencing and qPCR results (Fig 10). In addition, E6 mutants that were unable to degrade NHERF1 failed to increase nuclear YAP (Fig 7). These results implicated NHERF1 as an important regulator of YAP activation.

As with the high-risk HPVs, low-risk HPVs are a group of evolutionarily successful viruses that can persist in the epithelium basal layer. During lesion maintenance, the high-risk infections may progress to neoplasia whereas the low-risk group causes significant mobility and healthcare burden, but usually only low-grade lesions [93,94]. The current treatments, which depend on repeat surgical resection of papillomatous disease does not address the fundamental underlying issue of chronic infection with low-risk HPV types, and does not facilitate clearance of the reservoir of infected cells [6,7,95]. Despite the different disease outcomes, it appears that low-risk and high-risk HPVs modulate similar pathways during lesion expansion and persistence. Successful establishment of persistent infection is a prerequisite for both low-risk HPV chronic lesion and high-risk HPV carcinogenesis [11]. Our work enhances our understanding of E6 function during HPV infection and the consequences of its association with particular cellular proteins. The specific roles of E6 in lesion maintenance that are identified here, may in the future help direct the development of therapeutic agents designed to disadvantage the infected cell by targeting the molecular pathways that it depends on for persistence in the epithelial basal layer.

## Materials and methods

### Ethics statement

This study was approved by the institutional review board (IRB, Helsinki Committee) of The Galilee Medical Center. Approval Number NHR 0202–18 on March 12, 2018. The study was determined by the IRB to be exempt from obtaining patients’ consents forms. The mode of collection, processing, and patient data-handling of the clinical samples used in this study have been described previously [96].

### Cell culture

NIKS (a gift from Paul Lambert, McArdle Laboratory for Cancer Research, University of Wisconsin), a HPV-negative spontaneously immortalised human keratinocyte cell line, was maintained at sub-confluence on γ-Irradiated J2 3T3 feeder cells (a gift from Paul Lambert) in F medium with all supplements as previously described [97]. 293T (ATCC) were maintained in Dulbecco’s Modified Eagle’s Medium (DMEM, SIGMA) supplemented with 10% fetal calf serum (FCS, HyClone) and 1% penicillin and streptomycin. FUCCI NIKS cells were established by transducing with the FUCCI cell cycle sensor and FACS sorted for high level expression of Cdt1-mKO2 (G1/G0 phase) and geminin-mAG (S/G2/M phase). E6AP KO or mock control cell lines were established by transfection of px459 with sgRNA targeting E6AP or a non-exist gene (S2 Table).

### Plasmid construction and site-directed mutagenesis

pSpCas9(BB)-2A-Puro (PX459)-E6APgRNA plasmid and pSpCas9(BB)-2A-Puro (PX459)-scrambled control gRNA plasmid were kind gifts from Lawrence banks [98] and Dr. Yongxu Lu from Department of Pathology, University of Cambridge. Construction of the retroviral vectors pQCXIN-Flag11E6 and pQCXIN-Flag16E6 were accomplished by cloning the coding sequence using Gateway Technology (Thermo Fisher Scientific, MA, USA) following manufacturer’s instructions. The E6 mutants pQCXIN-Flag11E6^W133R^, pQCXIN-Flag11E6^L111Q^, pQCXIN-Flag11E6^L70A^, pQCXIN-Flag16E6^L50G^, pQCXIN-Flag16E6^F69A^ were constructed using a KOD-Plus-Mutagenesis Kit (TOYOBO, Japan), prior to DNA sequencing to ensure that no additional base changes were present. The primer sequences used for mutagenesis are listed in S1 Table. The E6AP-specific shRNA construct pCL-SI-MSCVpuro-H1R-E6APRi4 was described previously [99]. pBOB-EF1-FastFUCCI-Puro was a gift from Kevin Brindle & Duncan Jodrell (Addgene plasmid 86849) [40].

### Retrovirus transduction

The production and infection of recombinant retroviruses were accomplished as previously described [100,101]. To generate NIKS cells expressing E6, 2x10^5^ cells were seeded in each well of a six-well plate the day before transduction. Cells were inoculated with viruses at MOI>1 in the presence of 4 μg/ml of Polybrene (Santa Cruz). Stable NIKS populations were generated following selection with puromycin (10 μg/ml), G418 (50 μg/ml) or hygromycin (10 μg/ml).

### Organotypic raft culture

Raft cultures were established as previously described [97,102]. EF-1F human foreskin fibroblasts were mixed at a concentration of 10^7^ cells/ml with Rat Tail Collagen Type I (SLS, 354236) to make the dermal equivalent. Dermal equivalent was allowed to contract in DMEM for four days before NIKS cells were plated on at a density of 1.5 x 10^6^ cells/50 μl. Organotypic rafts were firstly cultured in FC media to allow attachment and expansion, followed by cornification media [97] to facilitate the formation of cornified layer. Rafts were allowed to stratify for approximately 14 days, then trimmed and fixed in 4% paraformaldehyde (PFA) for 24 hours. Tissue sectioning was performed by histologist at Department of Pathology, Cambridge.

### Immunofluorescence

Immunofluorescence was performed as described previously [103]. The formalin fixed, paraffin embedded (FFPE) tissue sections were wax removed with Xylene and incubated in Target retrieval solution pH 9.0 (Dako, Glostrup, Denmark) for 10 minutes at room temperature prior to incubating for 15 minutes at 110°C. The sections or cell samples were washed in PBS and fixed in 4% paraformaldehyde (PFA) in PBS for 10 minutes at room temperature. Cells were permeabilised in PBS with 0.1% Triton X-100 (Promega) for 30 minutes, then washed in PBS. The sections or cells were blocked in 5% normal goat serum in PBS for 1 hour prior to incubation of the primary antibodies overnight. The antibodies used were listed in S3 Table. Antigen antibody complexes were visualised with anti-mouse Alexa 488- or 594-conjugated antibody (Thermo Fisher Scientific) or Immpress anti-mouse/rabbit coupled with tyramide amplification kit (PerkinElmer, Inc). Nuclei were counterstained with DAPI.

### RNA *in situ* hybridisation

Viral RNA in cells were detected and visualized with RNAscope in situ hybridization assay (Advanced Cell Diagnostics, MN, USA) following the manufacturer’s instructions. The probe used for low-risk E6/E7 RNA detection was RNAscope Probe-HPV6/11 (415211).

### FUCCI cell density and competition assays

NIKS cells stably expressing both FUCCI cell cycle indicator and E6 were plated in CellCarrier-96 well ultra Microplates (Perkin Elmer) at 10 different densities, ranging from 6000 cells/well to 60,000 cells/well. FC media was changed at 24 hours after seeding and cells were fixed at 72 hours after seeding using 4% PFA. For competition assay, NIKS were seeded at high (confluent) density in CellCarrier-96 well ultra Microplates. To each well, 2.4x10^4^ NIKS cells of each mCherry and eGFP were seeded with 6 x 10^3^ irradiated J2-3T3 feeder cells. Cells were cultured for up to 9 days and media was changed every other day. Cells were fixed at day 1, 3, 5, 7 and 9 by incubating in 4% PFA for 30 minutes. Bottom layer and second layer of the cells were visualised and scanned by Harmony Opera Phenix high content imaging system at MRC Institute of Metabolic Science (IMS), Cambridge. Magnification 20x, Field size 3.78 mm^2^.

### SDS-PAGE and Western blotting

Proteins were extracted from cells using RIPA buffer and quantified using the BCA protein assay kit (Pierce), before being separated on 4–12% gradient polyacrylamide-SDS-Tris-Tricine denaturing gel (Invitrogen) and transferred onto PVDF membranes (IPFL00010, Merck). After transfer, membranes were blocked for 1 hour at room temperature in 5% milk in TBS. Blots were then incubated overnight at 4°C with appropriate primary antibody diluted in 5% milk in TBS. This is followed by incubating with appropriate IRDye 800cW fluorescent secondary antibody (Licor) for an hour at room temperature. Protein bands were detected with Odissey imaging system (Licor). Primary antibodies used in this study are listed in S3 Table.

### RNA sequencing

Total RNA was extracted from three independent clones of NIKS parental control cells, NIKS-E6AP^-/-^, or NIKS transduced with E6 using the RNeasy mini kit (Qiagen). PolyA selection, reverse transcription, library construction, sequencing and bioinformatics analysis were performed by Novogene. Differentially expressed genes were selected based on a log2 (FoldChange) > = 1 & padj < = 0.05 cut-off and were analysed for enriched biological processes using the GO (Gene Ontology) enrichment analysis tool. FPKM (expected number of Fragments Per Kilobase of transcript sequence per Millions base pairs sequenced) values were calculated to estimate gene expression levels from RNA-seq results.

### qPCR and RT-qPCR

Genomic DNA from NIKS for qPCR was purified using QIAamp DNA Mini Kit (Qiagen), according to the manufacturer’s instructions. All samples were digested with DpnI to remove any residual input DNA prior to analysis. Total RNA from NIKS was purified by using the RNeasy Mini Kit (Qiagen), with genomic DNA removed by Turbo DNA-free kit (Invitrogen). cDNA was synthesised with SuperScript III Reverse Transcriptase (Thermo Fisher scientific) using 100 μM oligo dT, according to the manufacturer’s instructions. The YAP-responsive genes *PTGS2*, *PLAU*, *AREG*, *AXL*, *CTGF* and E6 gene and GAPDH were measured by ViiA 7 Real-Time PCR system (Life Technologies) using Fast SYBR master mix (Applied Biosystems) with 15 minutes denaturation at 95°C, followed by 45 cycles of 95°C for 15 seconds and 60°C for 60 seconds. The PCR primers for qPCR are listed in S1 Table.

### Image capture and analysis

Images of 9 fields in each well of the 96 well plate were captured by High content confocal imaging at x20 magnification (4 channels: DAPI (461nm), geminin (488nm), Cdt1 (594nm) and K10 (650nm). % geminin positive cells, number of cells and % K10 positive cells were quantified with Harmony image analysis software (Perkinelmer). Same thresholds were applied to all plates. Size of each field = 3.78 mm^2^.

## Supporting information

S1 Fig NIKS and primary NHEK cells display similar characteristics in monolayer culture. (A) NHEK cells or NIKS cells stably expressing either 11E6 or 16E6 were compared at similar densities for the expression level of MCM (green) and K10 (red). Scale bar = 200 μm (B) Graphs comparing the saturation density, cells in cycle (at saturation), and differentiation commitment at saturation among EV-transduced, 11E6 or 16E6 expressing NHEK and NIKS cell lines. Statistical significance was calculated with one-way ANOVA. *, P ≤0.05; ***, P ≤0.001; ****, P ≤0.0001.
(TIF)

S2 Fig E6 and HPV16 genome-expressing organotypic raft cultures produce similar phenotypes. (A) NIKS rafts stably expressing 16E6 or maintaining the HPV16 full genome were stained with K10 (Thermofisher, MA5-13705) and MCM (Abcam ab52489) and compared to NIKS-EV control raft. Scale bar = 200 μm. (B) Basal cell density, cells in cycle in the basal layer, and differentiation commitment were quantified by calculating the mean values of nuclei per mm, % MCM7-positive cells in the basal layer, and distance from the basal lamina to K10 positive cells (μm). Error bars representant standard errors. Statistical significance was calculated with one-way ANOVA. *, P ≤0.05; **, P ≤0.01; ****, P ≤0.0001.
(TIF)

S3 Fig Validation of NHERF1 degradation deficient mutants of E6. NIKS cells were retrovirally transduced with vectors encoding 11E6, 16E6, 11E6^K73A^, 11E6^L70A^, 16E6^K72A^ and 16E6^F69A^. Cells were cultured at 8x 10^6^ cells/well in six-well plates and cell lysates were collected for western blotting. NHERF1 (Santa Cruz, sc271552) and GAPDH (EMD Millipore Corp. USA, MAB374) primary antibodies were used to detect specific protein bands.
(TIF)

S4 Fig Protein and mRNA Expression level of E6 and E6 mutants. (A) E6 mRNA expression level in NIKS cell lines stably expressing wild type E6 was compared with qRT-PCR. Results for three independent samples for each cell line were included in the analysis. Error bar shows the standard error. (B) E6 protein levels from cell extracts are shown by western blot analysis. Positive control is a flag-tagged vaccinia protein C6 with similar size to E6. GAPDH is used as house-keeping gene.
(TIF)

S5 Fig Immunofluorescence staining of low-risk HPV infected human tissues with separated channels. E6/E7 RNAScope, MCM7, K10, E6AP and NHERF1 immunofluorescence stainings were carried out on adjacent sections. Nuclei were counterstained with DAPI (blue). Scale bar = 200 μm. Lower power images are shown on the left. Enlargement images of non-infected and lesion area are presented with separated channels. The dotted lines indicate the position of the basal layer.
(TIF)

S6 Fig Immunofluorescence staining of high-risk HPV infected human tissues. (A) H&E staining (A), E4/MCM (B), K13 (C), and K10 (D) immunofluorescence stainings were performed on adjacent sections of high-risk HPV-infected low-grade squamous intraepithelial lesions (LSILs). Nuclei were counterstained with DAPI (blue). Scale = 200 μm. Both low power images and enlargement images of non-infected and lesion area are presented. The dotted lines indicate the position of the basal layer. (E) The graphs show quantifications of basal cell density, cells in cycle and differentiation commitment. Mean values of nuclei per mm, % MCM positive cells in the basal layer, and distance from the basal lamina to K13-positive cells were calculated and displayed with standard errors. Student t tests were performed (****, P ≤0.0001).
(TIF)

S7 Fig E6 expression causes E6AP level reduction in NIKS. (A) NIKS cells expressing either 11E6 or 16E6 were lysed and subject to western blotting for E6AP (Merck). (B) NIKS cells expressing either 11E6 or 16E6 were used to establish organotypic rafts, followed by immunofluorescent staining with E6AP (Merck, E8655). and DAPI. Scale bar = 200 μm.
(TIF)

S8 Fig YAP nuclear localisation is elevated in low-risk HPV lesion. Three condyloma biopsies from different patients were stained with active YAP antibody (Abcam, ab205270). Nuclei were stained with DAPI. Non-infected and lesion tissue areas are presented with separated channels. Scale bar = 200 μm.
(TIF)

S9 Fig E6 requires E6AP to promote YAP nuclear localisation. NIKS cells stably expressing shRNA targeting E6AP were transduced with plasmids expressing either 11E6 or 16E6. Cells were fixed at post-confluence and stained with active YAP antibody (Abcam, ab205270). Scale bar = 200 μm.
(TIF)

S10 Fig Pan-specific plot of YAP and phosphorylated YAP protein level in NIKS cells at high cell density upon E6 expression. NIKS cells stably expressing 11E6, 16E6, 11E6^W133R^, 11E6^L111Q^ and 11E6^L70A^ were harvested at high cell density and analysed with western blotting. Specific bands were detected using total YAP (cell signalling), active YAP (Abcam) and Ser127 YAP (cell signalling) antibodies.
(TIF)

S11 Fig E6AP depletion leads to delay of K10 expression in NIKS rafts. NIKS cells were transduced with lentiviral vectors encoding shRNA targeting either luciferase or E6AP. After selection, NIKS rafts were established, followed by H&E (left) and immunofluorescent staining (right) with K10 and DAPI. Scale bar = 200 μm.
(TIF)

S12 Fig 11E6 or 16E6 possesses E6AP-independent functions upon E6AP loss. E6AP KO NIKS cell lines were transduced with plasmids expressing either 11E6 or 16E6 and seeded at different densities in 96 well plates. Levels of K10 were measured and compared among control NIKS cells transduced with scrambled gRNA plasmid, NIKS-E6AP^-/-^ expressing either 11E6 or 16E6 and NIKSE6AP^-/—^EV. Each dot on the graph shows the % K10 positive cells against cell density per field (3.78 mm^2^) for each cell line. The red arrow indicates E6AP-independent functions of E6.
(TIF)

S13 Fig Differential gene expression (DEG) and Gene Ontology (GO) enrichment analysis of RNA sequencing results for E6AP-/- and E6-expressing cells. (A) Total number of differentially expressed genes (DEG) (purple), number of up-regulated genes (blue) and number of down-regulated genes (red) in each experimental group. (B) Venn diagram shows the number of co-expressed genes between the samples. (C) The X-axis displays the selected gene ontology (GO) terms that are the most relevant and significant, which ranks from left to right according to degree of significance. The y-axis shows the number of genes that were up-regulated (blue) or down-regulated (red) under each GO term.
(TIF)

S14 Fig HPV16 genome cannot be maintained in E6AP-/- cells. Genomic DNA was extracted from NIKS E6AP^-/-^ or control cells transfected with either HPV16 or HPV11 genome over 5 passages. The viral genome copy number per cell was measured by qPCR, using GAPDH to estimate cell number. Three biological repeats were included for each passage. Error bar indicates standard errors across three samples.
(TIF)

S1 Table Primers used in this study. (XLSX)

S2 Table Plasmids used in this study. (XLSX)

S3 Table Antibodies used in this study. (XLSX)

S1 File Differential expression gene clustering heatmap comparing 11E6-, 16E6-expressing and control NIKS. (PDF)

S2 File Differential expression gene clustering heatmap comparing E6AP^-/-^ and control NIKS. (PDF)
