# Supplementary figures and images for: HPV E6 inhibits E6AP to regulate epithelial homeostasis by modulating keratinocyte differentiation commitment and YAP1 activation
Source: PLoS Pathog. 2023 Jun 28;19(6):e1011464. doi: 10.1371/journal.ppat.1011464 (PMC10335691; doi:10.1371/journal.ppat.1011464)

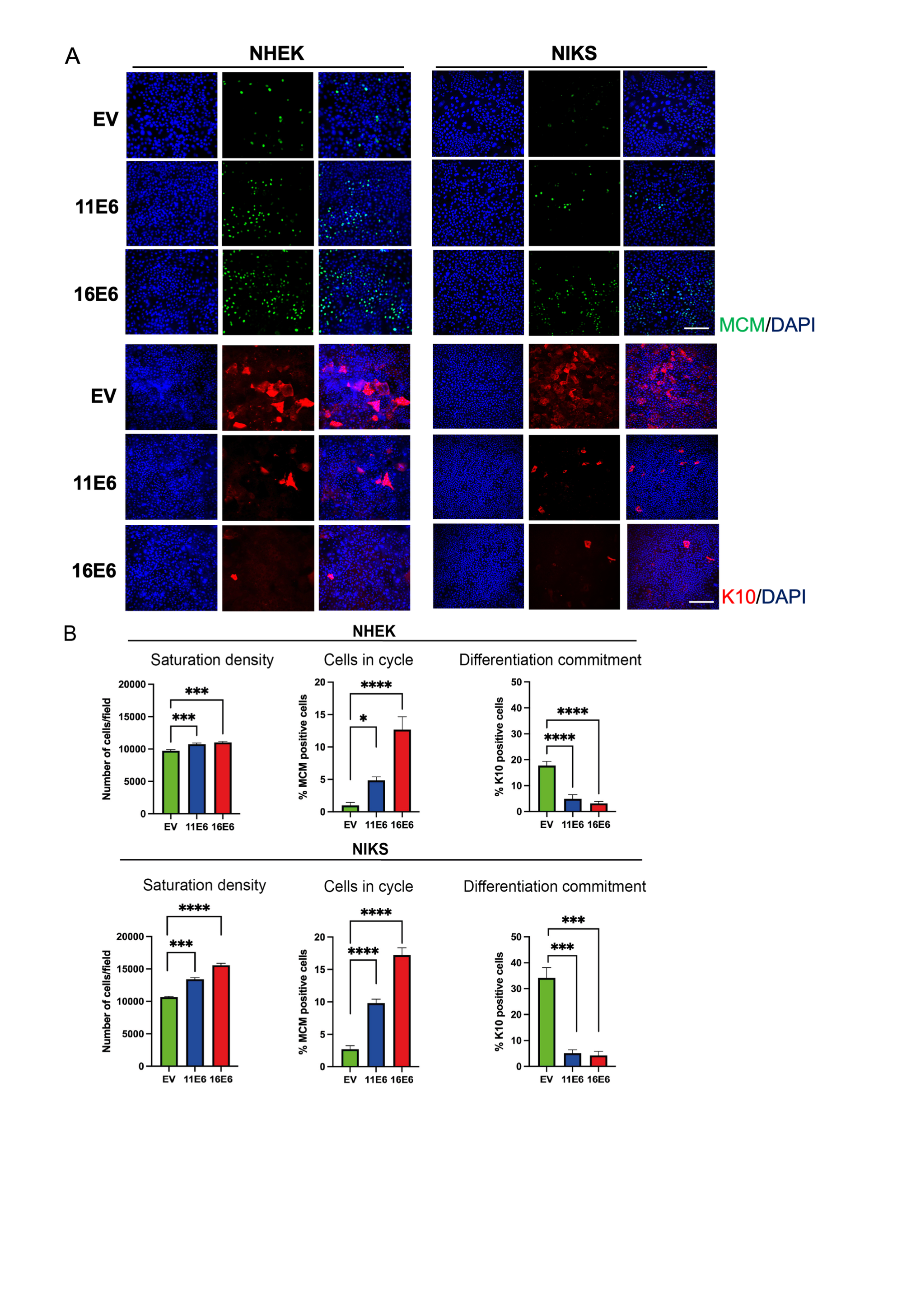

Supplement: S1 Fig — (A) NHEK cells or NIKS cells stably expressing either 11E6 or 16E6 were compared at similar densities for the expression level of MCM (green) and K10 (red). Scale bar = 200 μm (B) Graphs comparing the saturation density, cells in cycle (at saturation), and differentiation commitment at saturation among EV-transduced, 11E6 or 16E6 expressing NHEK and NIKS cell lines. Statistical significance was calculated with one-way ANOVA. *, P ≤0.05; ***, P ≤0.001; ****, P ≤0.0001. (TIF) [file ppat.1011464.s001.tif]

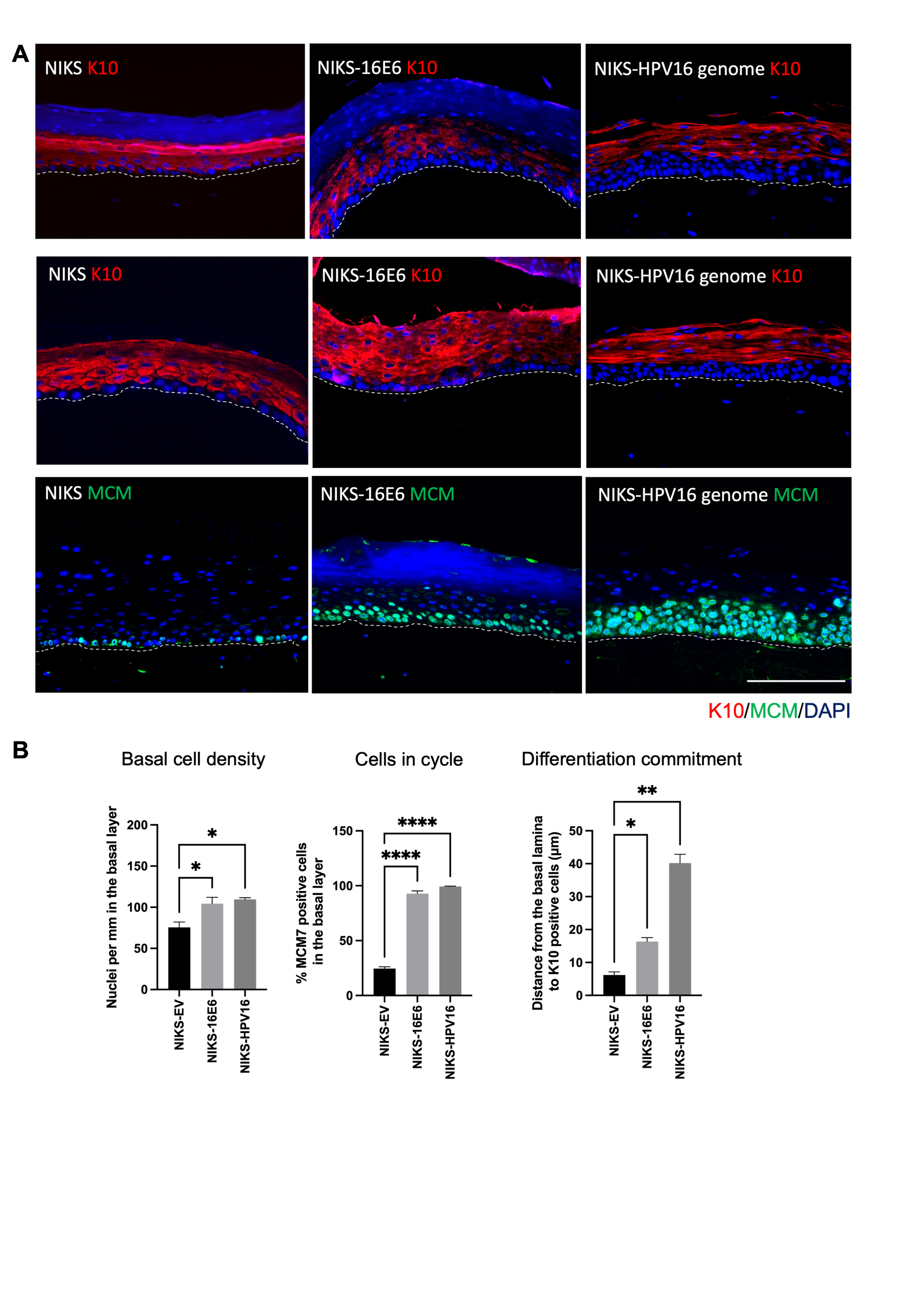

Supplement: S2 Fig — (A) NIKS rafts stably expressing 16E6 or maintaining the HPV16 full genome were stained with K10 (Thermofisher, MA5-13705) and MCM (Abcam ab52489) and compared to NIKS-EV control raft. Scale bar = 200 μm. (B) Basal cell density, cells in cycle in the basal layer, and differentiation commitment were quantified by calculating the mean values of nuclei per mm, % MCM7-positive cells in the basal layer, and distance from the basal lamina to K10 positive cells (μm). Error bars representant standard errors. Statistical significance was calculated with one-way ANOVA. *, P ≤0.05; **, P ≤0.01; ****, P ≤0.0001. (TIF) [file ppat.1011464.s002.tif]

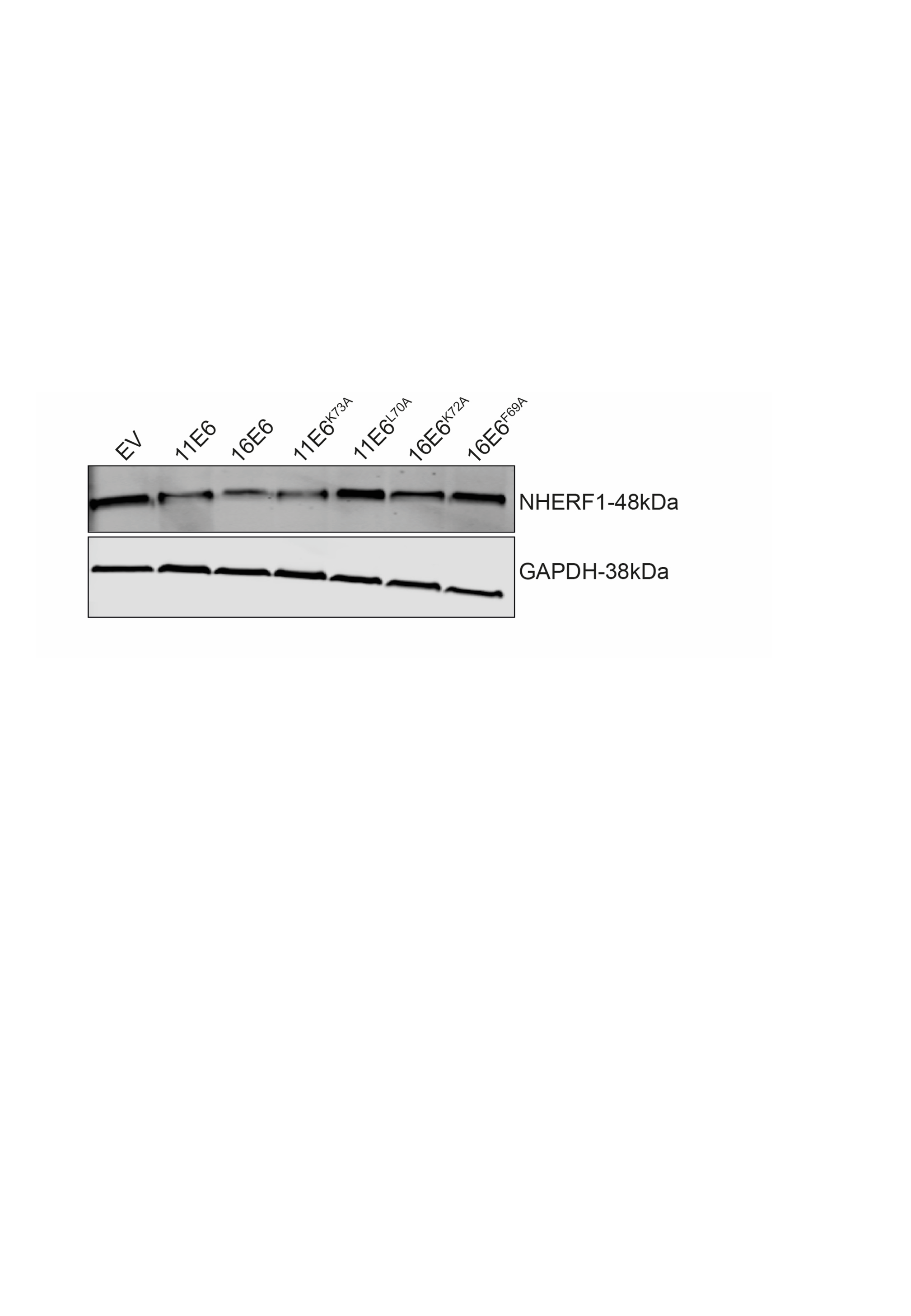

Supplement: S3 Fig — NIKS cells were retrovirally transduced with vectors encoding 11E6, 16E6, 11E6K73A, 11E6L70A, 16E6K72A and 16E6F69A. Cells were cultured at 8x 10^6^ cells/well in six-well plates and cell lysates were collected for western blotting. NHERF1 (Santa Cruz, sc271552) and GAPDH (EMD Millipore Corp. USA, MAB374) primary antibodies were used to detect specific protein bands. (TIF) [file ppat.1011464.s003.tif]

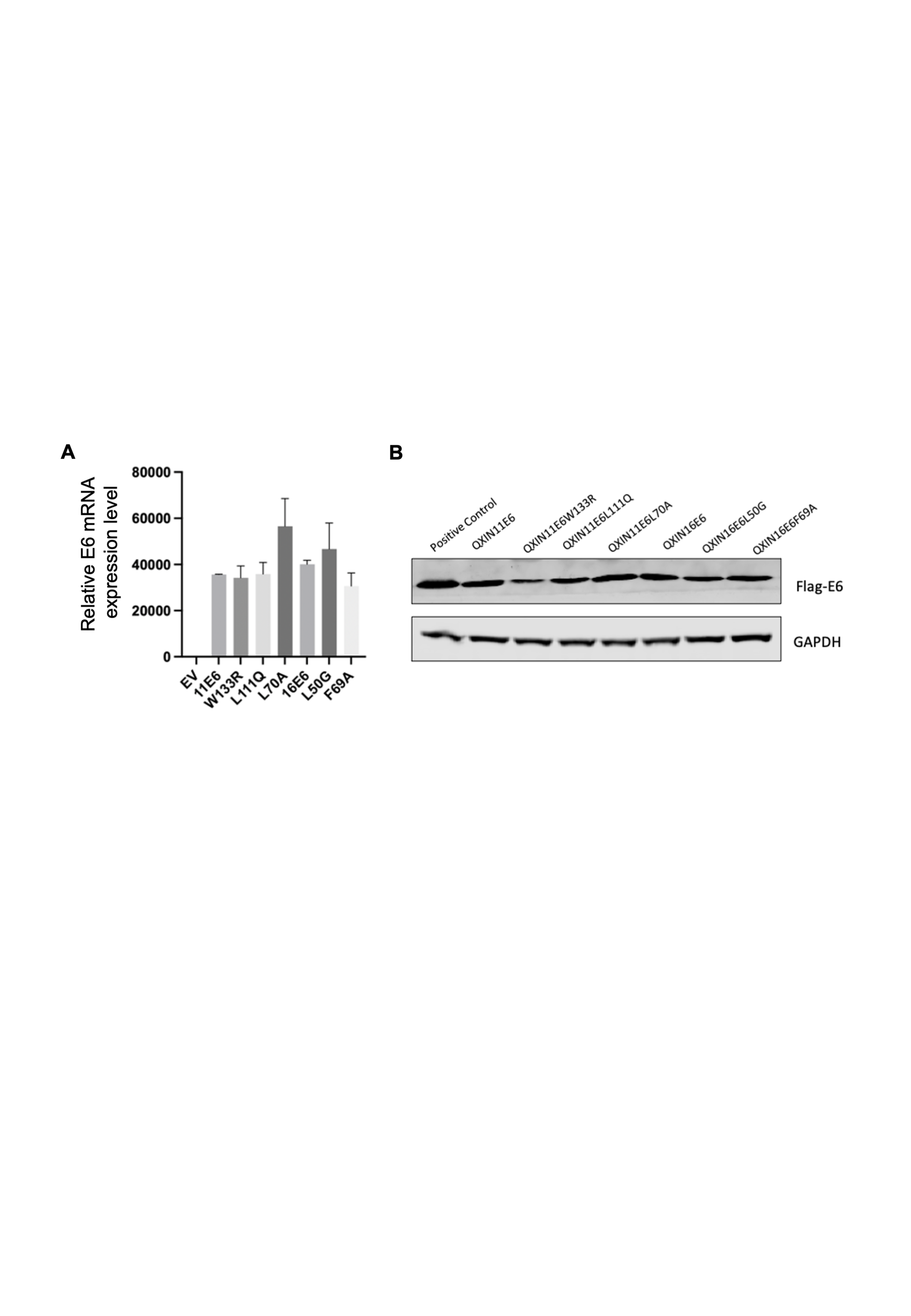

Supplement: S4 Fig — (A) E6 mRNA expression level in NIKS cell lines stably expressing wild type E6 was compared with qRT-PCR. Results for three independent samples for each cell line were included in the analysis. Error bar shows the standard error. (B) E6 protein levels from cell extracts are shown by western blot analysis. Positive control is a flag-tagged vaccinia protein C6 with similar size to E6. GAPDH is used as house-keeping gene. (TIF) [file ppat.1011464.s004.tif]

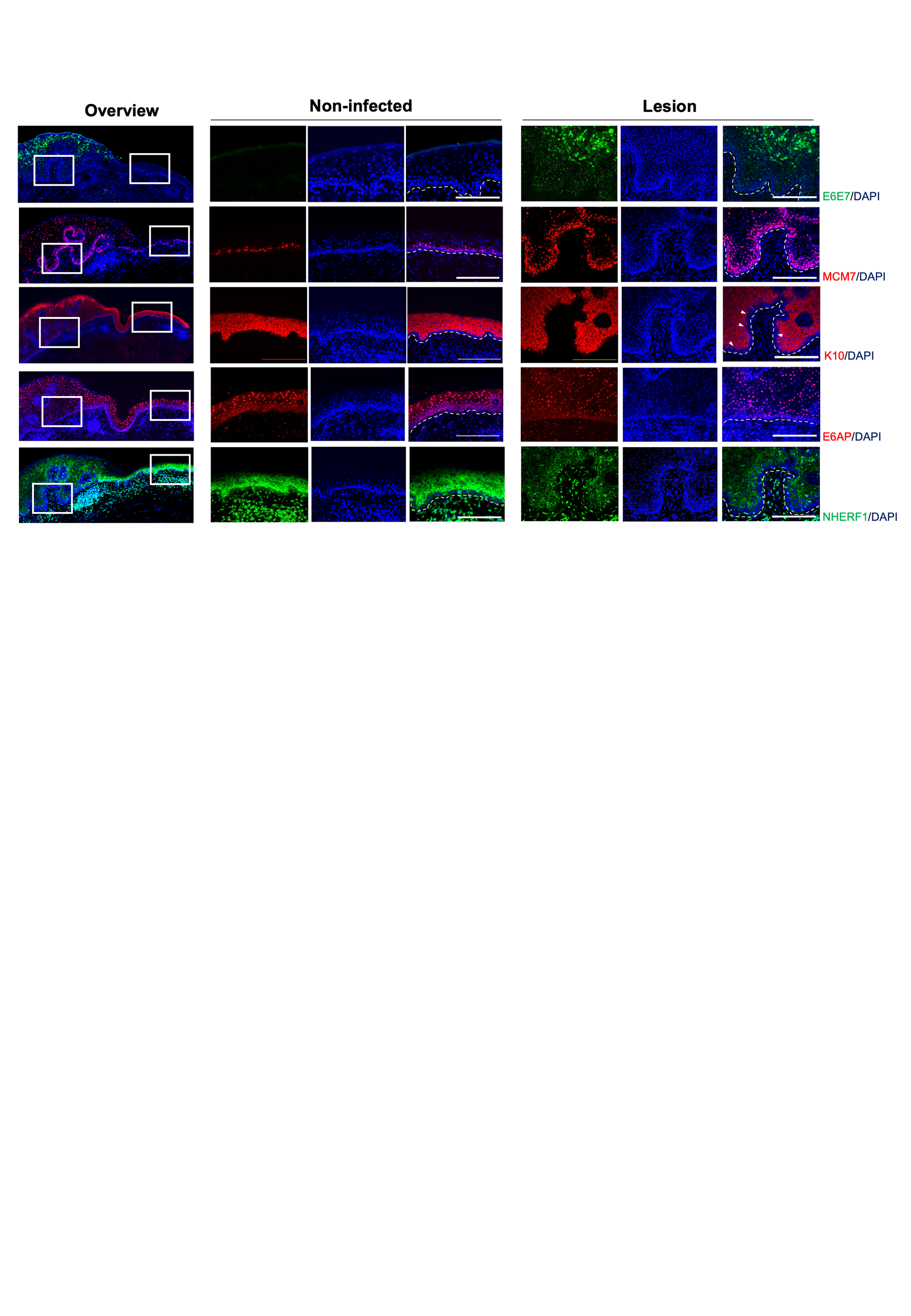

Supplement: S5 Fig — E6/E7 RNAScope, MCM7, K10, E6AP and NHERF1 immunofluorescence stainings were carried out on adjacent sections. Nuclei were counterstained with DAPI (blue). Scale bar = 200 μm. Lower power images are shown on the left. Enlargement images of non-infected and lesion area are presented with separated channels. The dotted lines indicate the position of the basal layer. (TIF) [file ppat.1011464.s005.tif]

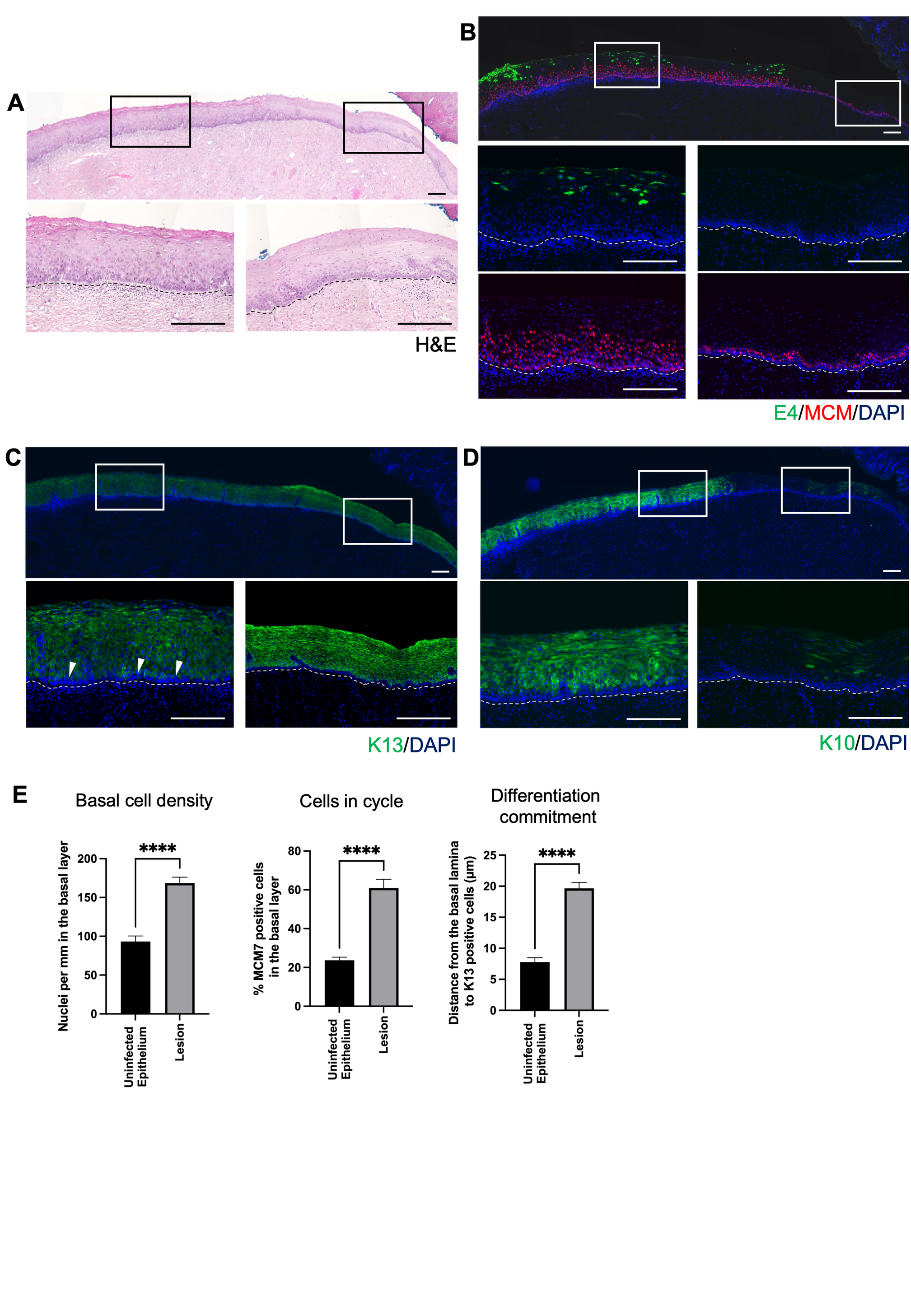

Supplement: S6 Fig — (A) H&E staining (A), E4/MCM (B), K13 (C), and K10 (D) immunofluorescence stainings were performed on adjacent sections of high-risk HPV-infected low-grade squamous intraepithelial lesions (LSILs). Nuclei were counterstained with DAPI (blue). Scale = 200 μm. Both low power images and enlargement images of non-infected and lesion area are presented. The dotted lines indicate the position of the basal layer. (E) The graphs show quantifications of basal cell density, cells in cycle and differentiation commitment. Mean values of nuclei per mm, % MCM positive cells in the basal layer, and distance from the basal lamina to K13-positive cells were calculated and displayed with standard errors. Student t tests were performed (****, P ≤0.0001). (TIF) [file ppat.1011464.s006.tif]

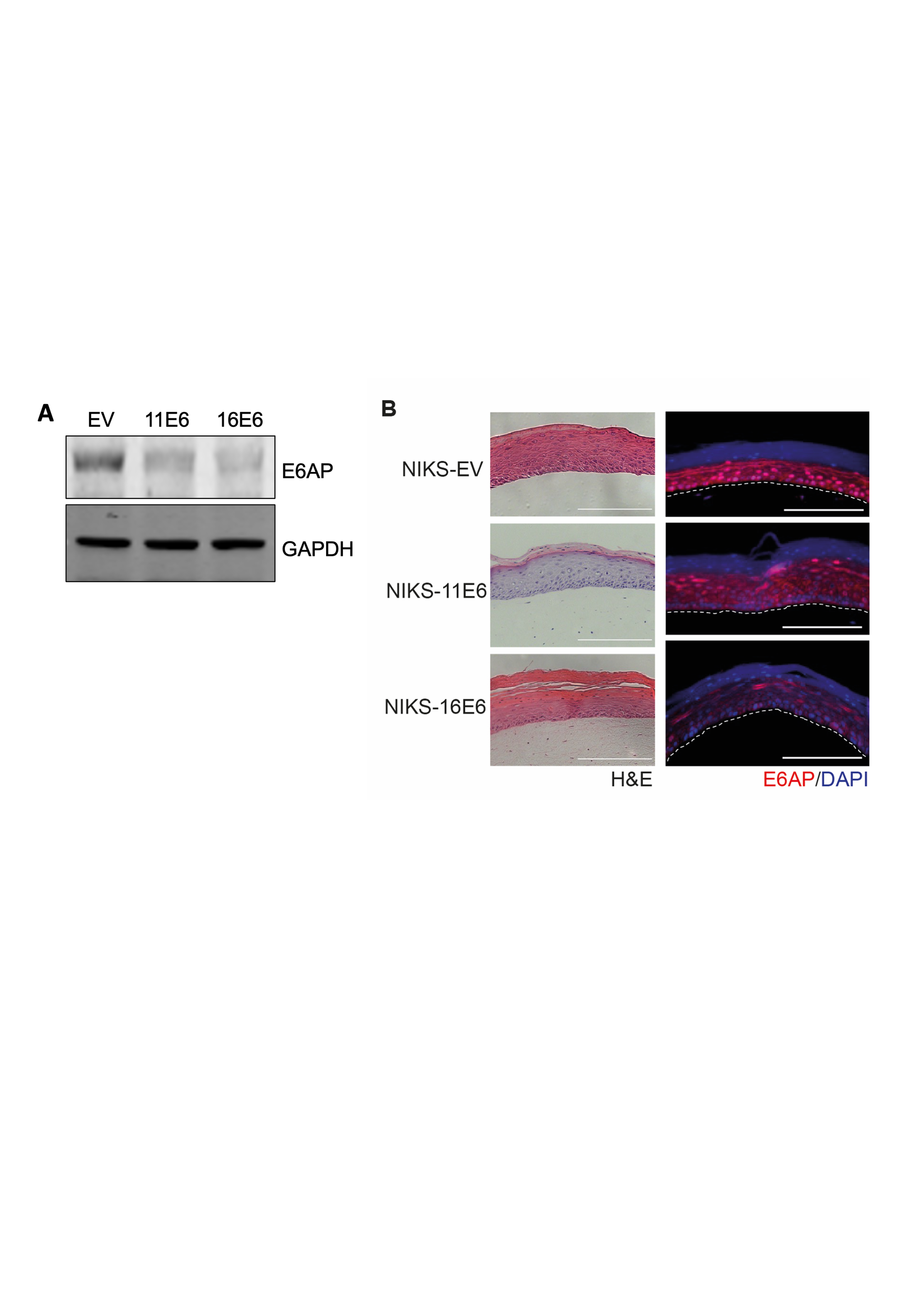

Supplement: S7 Fig — (A) NIKS cells expressing either 11E6 or 16E6 were lysed and subject to western blotting for E6AP (Merck). (B) NIKS cells expressing either 11E6 or 16E6 were used to establish organotypic rafts, followed by immunofluorescent staining with E6AP (Merck, E8655). and DAPI. Scale bar = 200 μm. (TIF) [file ppat.1011464.s007.tif]

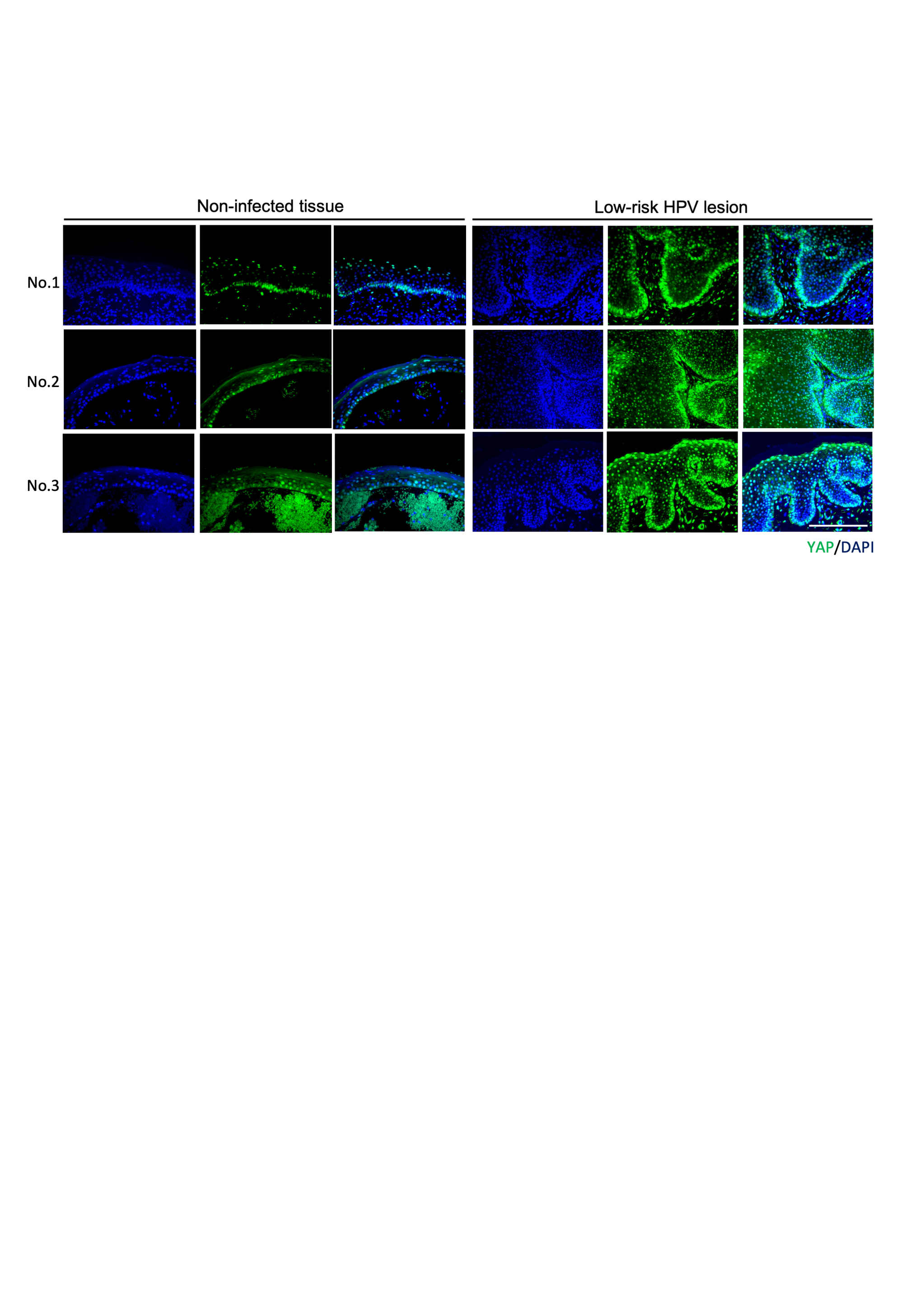

Supplement: S8 Fig — Three condyloma biopsies from different patients were stained with active YAP antibody (Abcam, ab205270). Nuclei were stained with DAPI. Non-infected and lesion tissue areas are presented with separated channels. Scale bar = 200 μm. (TIF) [file ppat.1011464.s008.tif]

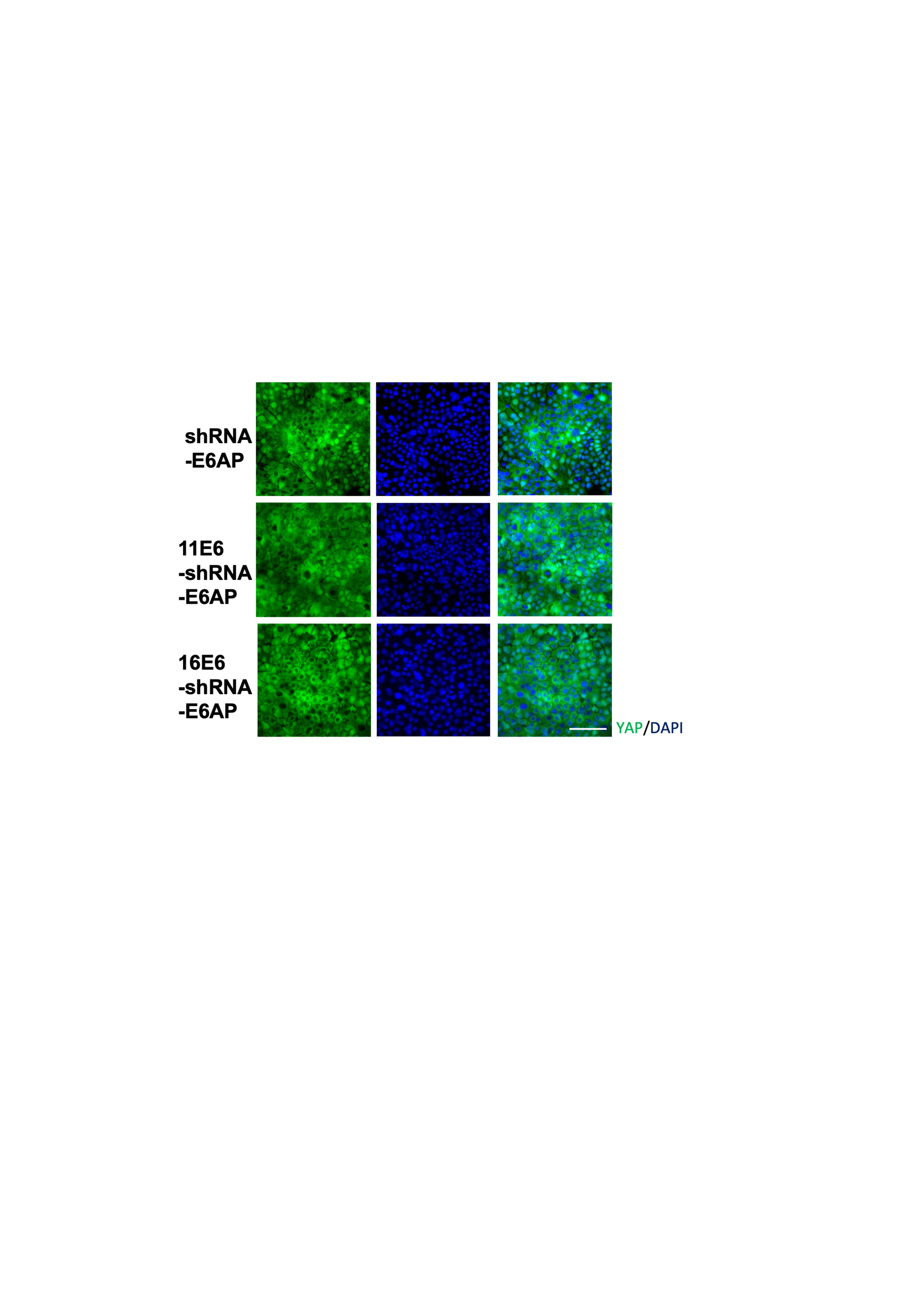

Supplement: S9 Fig — NIKS cells stably expressing shRNA targeting E6AP were transduced with plasmids expressing either 11E6 or 16E6. Cells were fixed at post-confluence and stained with active YAP antibody (Abcam, ab205270). Scale bar = 200 μm. (TIF) [file ppat.1011464.s009.tif]

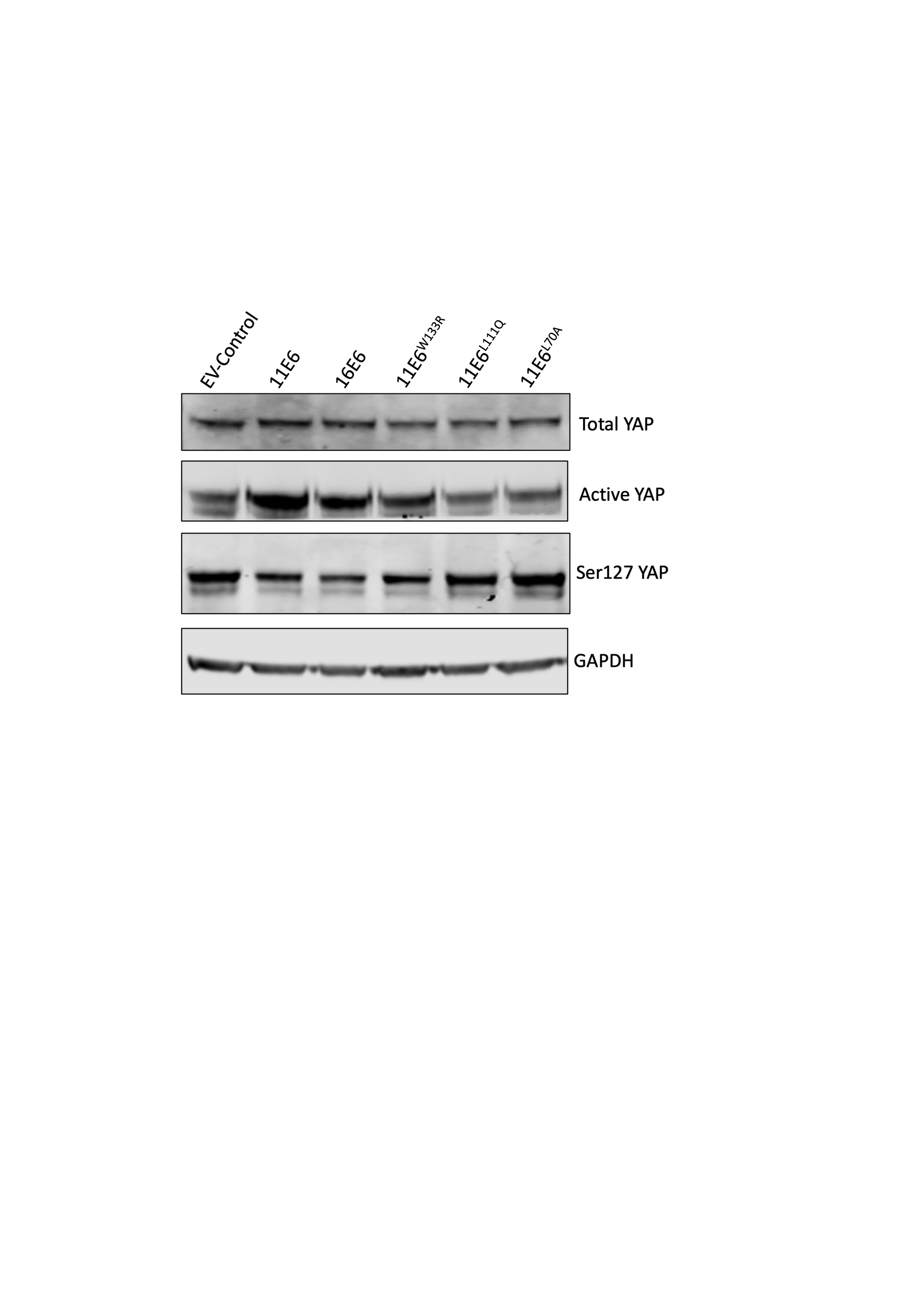

Supplement: S10 Fig — NIKS cells stably expressing 11E6, 16E6, 11E6W133R, 11E6L111Q and 11E6L70A were harvested at high cell density and analysed with western blotting. Specific bands were detected using total YAP (cell signalling), active YAP (Abcam) and Ser127 YAP (cell signalling) antibodies. (TIF) [file ppat.1011464.s010.tif]

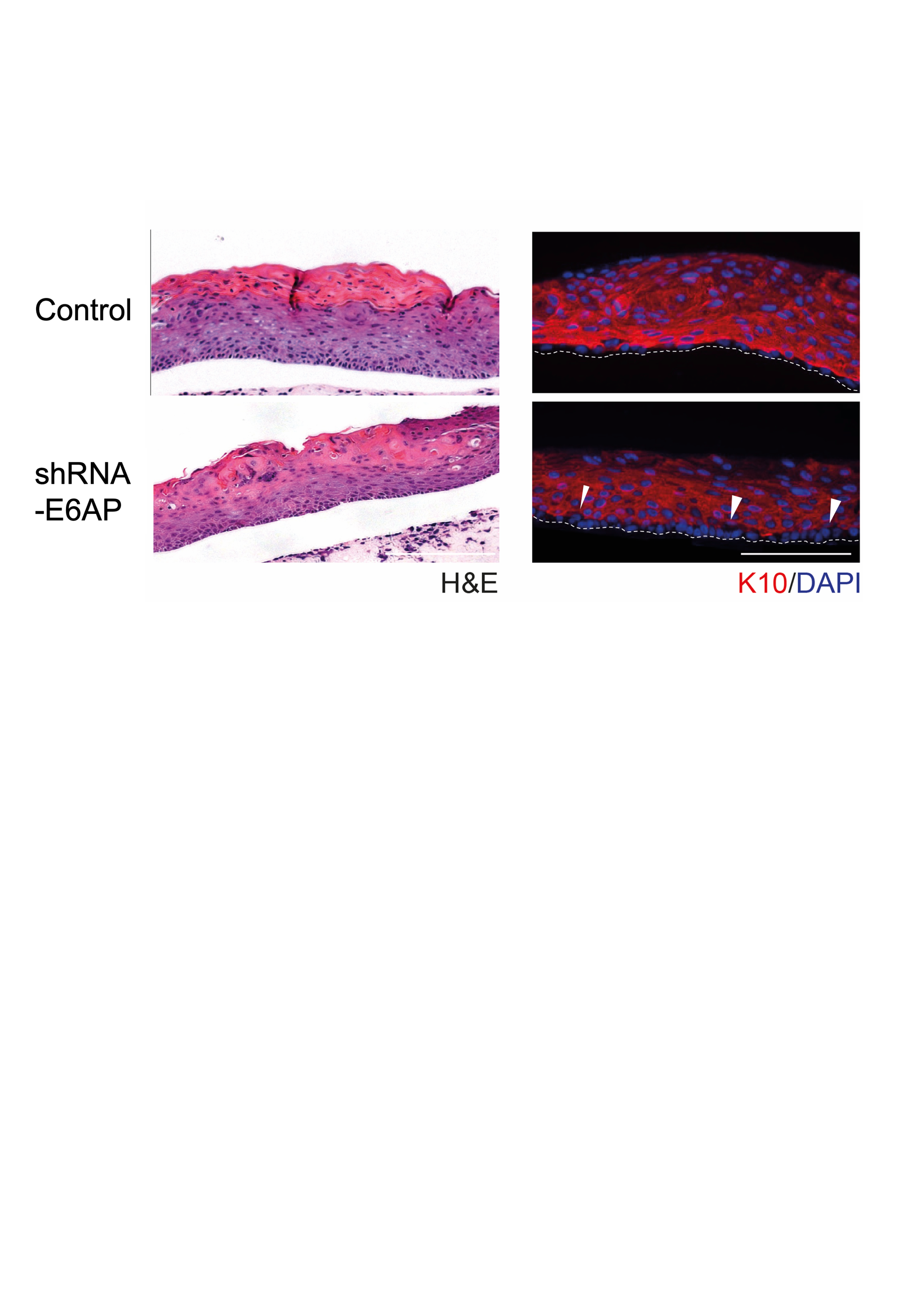

Supplement: S11 Fig — NIKS cells were transduced with lentiviral vectors encoding shRNA targeting either luciferase or E6AP. After selection, NIKS rafts were established, followed by H&E (left) and immunofluorescent staining (right) with K10 and DAPI. Scale bar = 200 μm. (TIF) [file ppat.1011464.s011.tif]

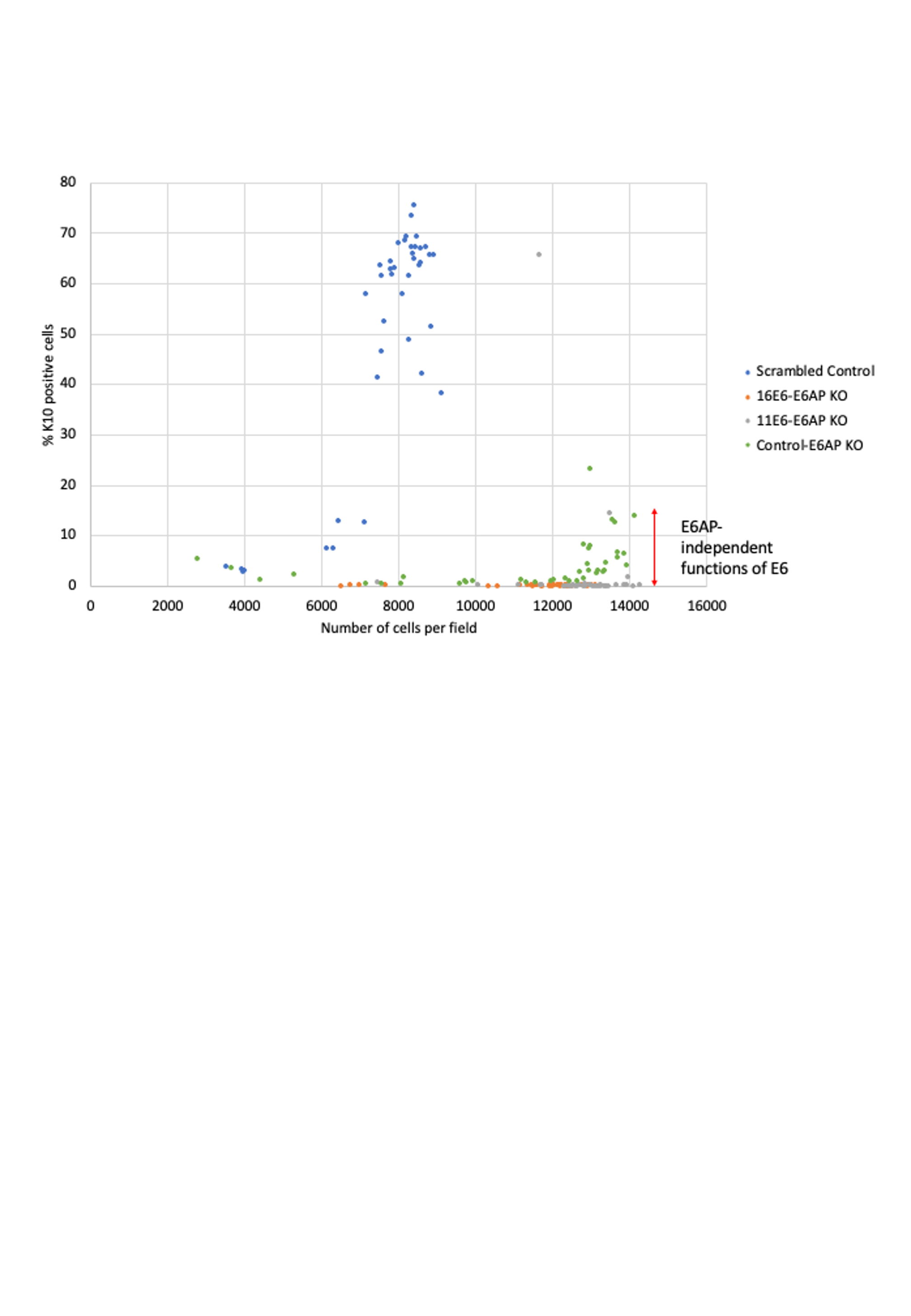

Supplement: S12 Fig — E6AP KO NIKS cell lines were transduced with plasmids expressing either 11E6 or 16E6 and seeded at different densities in 96 well plates. Levels of K10 were measured and compared among control NIKS cells transduced with scrambled gRNA plasmid, NIKS-E6AP-/- expressing either 11E6 or 16E6 and NIKSE6AP-/—EV. Each dot on the graph shows the % K10 positive cells against cell density per field (3.78 mm2) for each cell line. The red arrow indicates E6AP-independent functions of E6. (TIF) [file ppat.1011464.s012.tif]

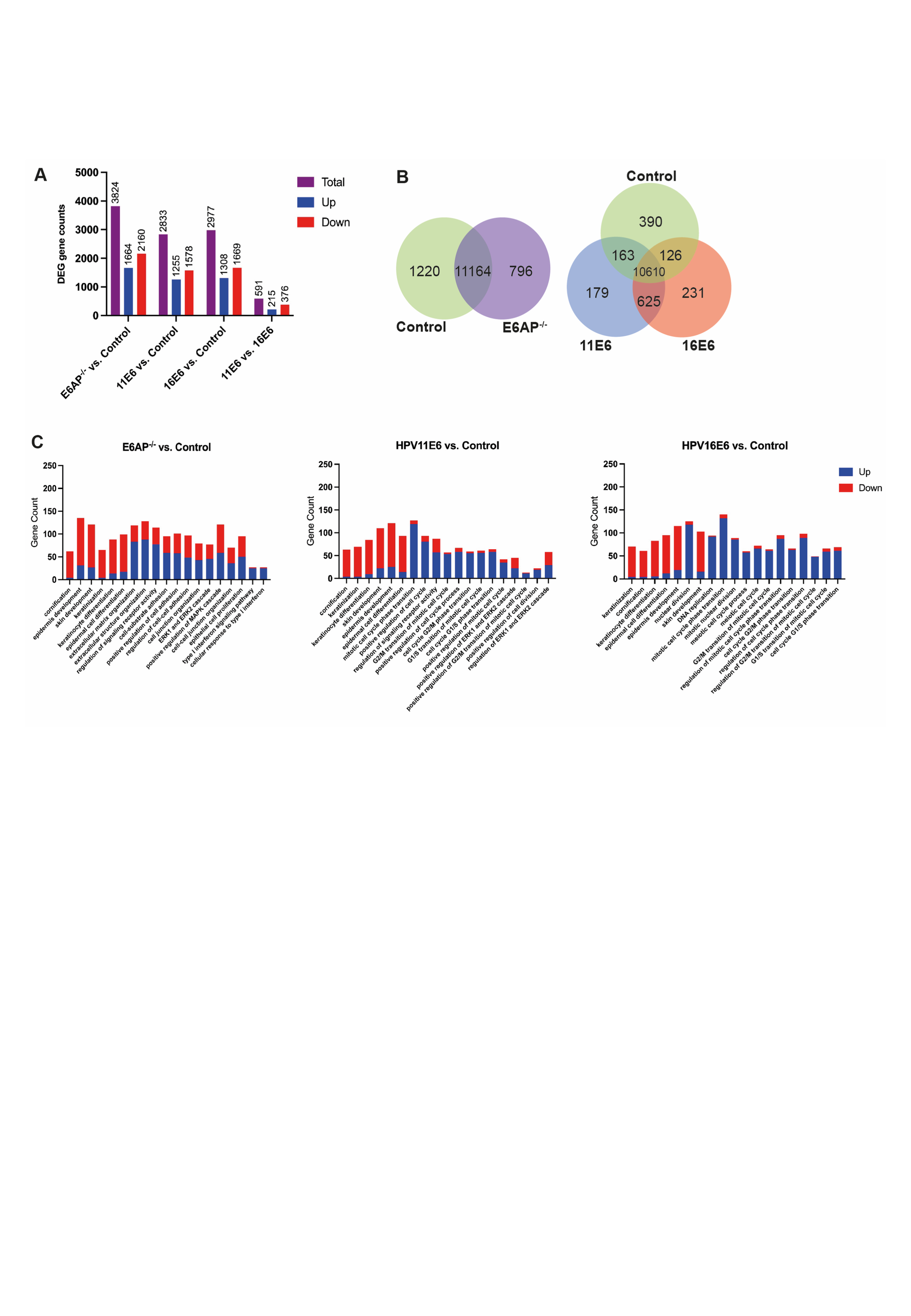

Supplement: S13 Fig — (A) Total number of differentially expressed genes (DEG) (purple), number of up-regulated genes (blue) and number of down-regulated genes (red) in each experimental group. (B) Venn diagram shows the number of co-expressed genes between the samples. (C) The X-axis displays the selected gene ontology (GO) terms that are the most relevant and significant, which ranks from left to right according to degree of significance. The y-axis shows the number of genes that were up-regulated (blue) or down-regulated (red) under each GO term. (TIF) [file ppat.1011464.s013.tif]

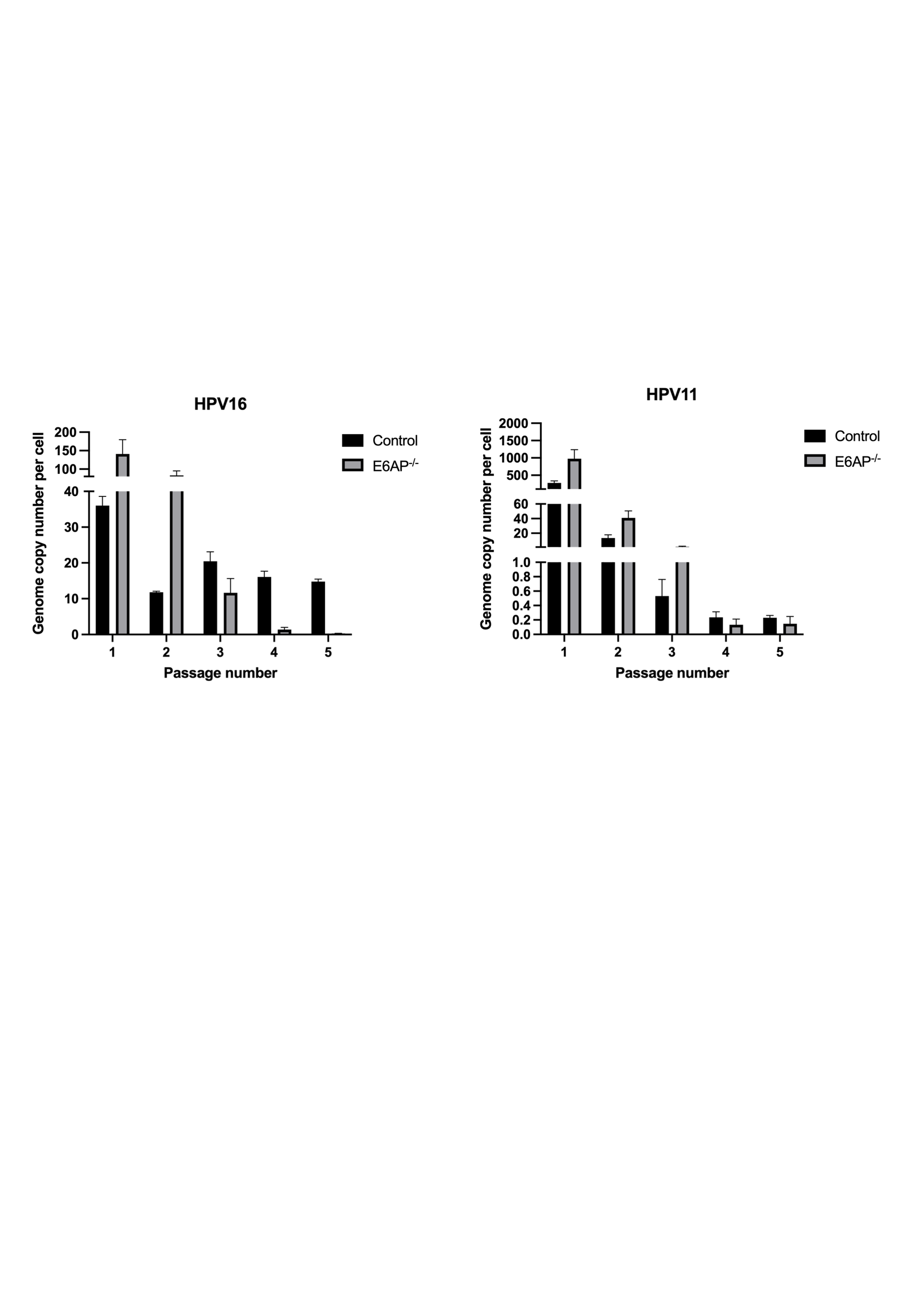

Supplement: S14 Fig — Genomic DNA was extracted from NIKS E6AP-/- or control cells transfected with either HPV16 or HPV11 genome over 5 passages. The viral genome copy number per cell was measured by qPCR, using GAPDH to estimate cell number. Three biological repeats were included for each passage. Error bar indicates standard errors across three samples. (TIF) [file ppat.1011464.s014.tif]
