# Supplementary material for: HPV E6 inhibits E6AP to regulate epithelial homeostasis by modulating keratinocyte differentiation commitment and YAP1 activation
Source: PLoS Pathog. 2023 Jun 28;19(6):e1011464. doi: 10.1371/journal.ppat.1011464 (PMC10335691; doi:10.1371/journal.ppat.1011464)

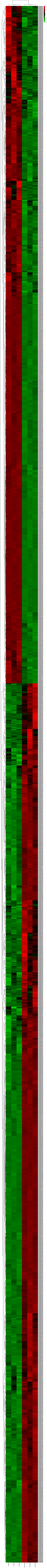

Supplement: S2 File — (PDF) [file ppat.1011464.s019.pdf]
